# Low androgen signaling rescues genome integrity with innate immune response by reducing fertility in humans

**DOI:** 10.1038/s41419-023-06397-5

**Published:** 2024-01-11

**Authors:** J. Zimmer, L. Mueller, P. Frank-Herrmann, J. Rehnitz, J. E. Dietrich, M. Bettendorf, T. Strowitzki, M. Krivega

**Affiliations:** 1 Research Group of Gonadal Differentiation and Embryonic Development, Department of Gynecological Endocrinology & Fertility Disorders, Women Hospital, University of Heidelberg, 69120 Heidelberg, Germany; 2 Department of Gynecological Endocrinology & Fertility Disorders, Women Hospital, University of Heidelberg, 69120 Heidelberg, Germany; 3 Division of Pediatric Endocrinology, Children’s Hospital, University of Heidelberg, 69120 Heidelberg, Germany

**Keywords:** Germ cell tumours, Cancer genomics, Apoptosis, Embryology, Spermatogenesis

## Abstract

Development of the gonads under complex androgen regulation is critical for germ cells specification. In this work we addressed the relationship between androgens and genomic integrity determining human fertility. We used different study groups: individuals with Differences of Sex Development (DSD), including Complete Androgen Insensitivity Syndrome (CAIS) due to mutated androgen receptor (AR), and men with idiopathic nonobstructive azoospermia. Both showed genome integrity status influenced by androgen signaling via innate immune response activation in blood and gonads. Whole proteome analysis connected low AR to interleukin-specific gene expression, while compromised genome stability and tumorigenesis were also supported by interferons. AR expression was associated with predominant DNA damage phenotype, that eliminated AR-positive Sertoli cells as the degeneration of gonads increased. Low AR contributed to resistance from the inhibition of DNA repair in primary leukocytes. Downregulation of androgen promoted apoptosis and specific innate immune response with higher susceptibility in cells carrying genomic instability.

## Introduction

Gender-specified differentiation of the bipotential gonad is tightly regulated by hormonal balance [1, 2]. Follicle-stimulating hormone (FSH) and luteinizing hormone (LH) are regulators of oogenesis, while testosterone with FSH promotes spermatogenesis [3, 4]. Disbalance of these hormones can lead to germ cell apoptosis [5, 6]. Low androgens are the main cause of defective spermatogenesis, while elevated levels of androgen are characteristic for polycystic ovarian syndrome [7, 8]. The androgen receptor (AR) gene is encoded on X chromosome and can carry hundreds of mutations causing Complete Androgen Insensitivity Syndrome (CAIS) [9, 10]. The presence of absence of multiple genetic abnormalities in the AR gene show limited correlation with physiological phenotypes [11, 12]. Mutations in noncoding regions of the AR gene or other currently unidentified genetic alterations unrelated to AR might also cause CAIS [13]. All of this questions the necessity of genetic diagnostics of CAIS, and rather emphasizes importance of characterization of general molecular mechanisms that could be responsible for the pathological phenotypes due to the androgen imbalance.

Patients with CAIS belong to the group of individuals diagnosed with Differences of Sex Development (DSD), associated with dysgenic gonad formation, germ cells tumor (GCT) development, and therefore infertile. Individuals with CAIS are usually women with male genotype (46, XY) that does not develop due to the non-functional androgens. Other individuals e.g., women with Swyer (46, XY) syndrome also belong to the DSD group, and their disease phenotypes were recently linked to the genomic instability [14]. Compromised DNA repair mechanisms were linked to a significant risk of GCT development, particularly, in AIS that increases with age to about 20-50% [14, 15]. Therefore, individuals with CAIS are often undergoing gonadectomy before puberty. The following changes in hormonal status complicate the gender specification. Keeping the gonads may be also beneficial if resilient germ cells in the gonads can be obtained for IVF procedures [16, 17].

Previously we showed that most of the individuals with DSD carry increased DNA damage in blood and expose genotoxic stress phenotypes in dysgenic gonadal tissue [14]. Similar genotoxic stress phenotype was characterized by increased innate immune responses in other cells [18, 19]. Interestingly, individuals with CAIS did not show increased DNA damage in peripheral blood samples and expose a rather mild phenotype in the gonads [14]. However, similar to the blood samples from individuals with GCT, they upregulate interferon beta (*IFNβ*) and interleukin 6 (*IL6*) together with a wide range of innate immune-relevant response proteome. It is however unclear whether innate immune response upregulation in CAIS group could be partially attributed to the downregulation of AR [20]. It is known that inhibited AR led to DNA damage via inflammatory STING activation in melanoma cells, which was beneficial for patient survival due to interferon signaling and improved DNA repair mechanisms [21]. On the other hand, women with hyperandrogenism exhibit disbalance of immune cells leading to low-grade but chronic inflammation [22]. Further investigation is needed to determine the potential relationship between AR downregulation and the observed upregulation of innate immune response genes.

Despite the discovery of hundreds de novo mutations, the underlying genetic causes for the majority of male infertility cases remain unknown [23–25]. Similar to individuals with DSD, elevated DNA damage and associated chronic inflammation are general infertility phenotypes in the blood and testes of men with idiopathic germ cell aplasia [26]. Importantly, a general increase in genetic mutations, that also includes genes of DNA damage response (DDR) is common among men with azoospermia [27–32], including Sertoli cell-only phenotype (SCO) [33, 34], resembling dysgenic gonads of CAIS individuals. Multiple DNA mutations observed in infertile men indicate genetic heterogeneity, which could potentially be attributed to genomic instability potentially similar to those in DSD- individuals. All these phenotypes potentially indicate a presence of similar molecular mechanisms in patients with different infertility diagnosis.

The important question is whether failed gonadal differentiation possibly due to increased DNA damage is connected to increasing risk of germ cell tumors. We hypothesized that inhibited AR signaling with its ability to sustain innate immune response reduces genotoxic stress phenotypes. Therefore, in this paper we aimed to analyze compromised genome integrity of individuals with DSD and nonobstructive azoospermia (NOA) in connection with androgen and innate immune response. We show that upregulated innate immune response due to low AR could play a protective role against raising genotoxic stress in individuals with dysgenic gonads and NOA, therefore potentially diminishing the risk of DNA mutagenesis and germ cell tumorigenesis, but simultaneously compromising gonadal and germ cell differentiation. A better understanding of molecular mechanisms of infertility will help to propose new therapeutical treatments and facilitate early germ cell tumor diagnostics, thus avoiding prophylactic gonadectomy.

## Results

### Deregulated innate immune response is associated with decreased AR

**Fig. 1:**
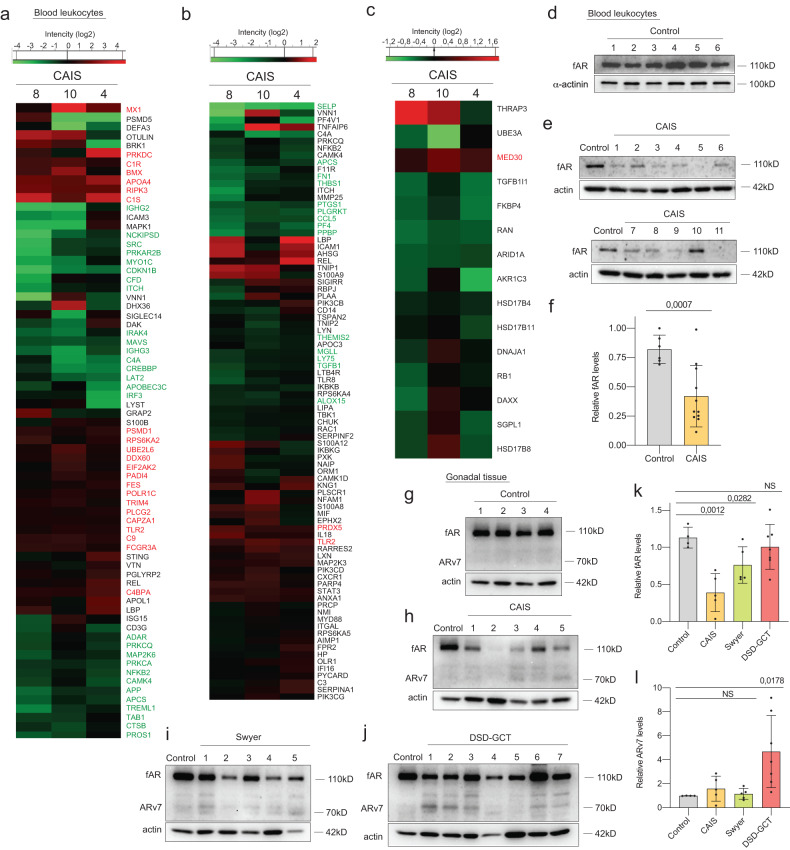
Innate immune response interconnection with AR signaling in blood from individuals with CAIS and in dysgenic gonads. Whole proteome analysis of leukocytes from blood of individuals with CAIS with GO for innate immune response (**a**), GO for inflammation (**b**) and GO for AR signaling (**c**). Immunoblotting for AR protein in blood samples of the control (**d**) and CAIS (**e**) groups and its quantification (**f**). Actin was used as the loading control. Immunoblotting for AR protein in gonadal tissue samples from the control (**g**), CAIS (**h**), Swyer (**i**) and DSD-GCT (**j**) groups. Quantification of fAR (**k**) and ARv7 (**l**) relative protein levels of the immunoblots (**g–j**). ANOVA and unpaired t test were used for statistical analysis; *p* values, means and standard deviations are shown on the plots.

Whole proteome analysis revealed variable changes in innate immune response in leukocytes from individuals with CAIS (Fig. 1a, Supplementary dataset 1, Supplementary table 1). Among these changes, DDR proteins such as MX1, PRKDC, BMX and APOA4 were upregulated [35–37]. Interestingly, the proteome related to inflammation was predominantly inhibited in CAIS, and there were no proteins consistently upregulated in all samples (Fig. 1b, Supplementary dataset 2), while proteome associated with AR signaling was mostly inhibited (Fig. 1c, Supplementary dataset 3). We did not observe any significant changes in *AR* transcript expression, while AR protein levels were downregulated and even undetectable in some CAIS samples, when compared to the control group (Fig. 1d-f; Extended data Fig. 1a). Intriguingly, *AR* RNA was found to be exceptionally elevated in blood of individuals with Swyer syndrome, and was also associated with GCTs in gonadal tissue (Extended data Fig. 1a, b, Supplementary table 1). Notably, we observed only the full-length form of AR protein (fAR) in blood samples, whereas in gonadal tissue, an additional shorter isoform of AR (ARv7) was present. fAR was particularly inhibited in gonads from individuals with CAIS (Fig. 1g-k, Supplementary Table 2), while relative protein levels of ARv7 were significantly induced by five-fold in tissues with GCT (Fig. 1l). Therefore, a deregulated innate immune response in blood correlates with the decreased AR signaling in both blood and gonads of individuals with CAIS. Furthermore, only the shorter isoform ARv7, along with uniquely upregulated *AR* transcripts, showed an association with GCTs.

### Specific interferon and interleukin signaling changes in individuals with DSD

**Fig. 2:**
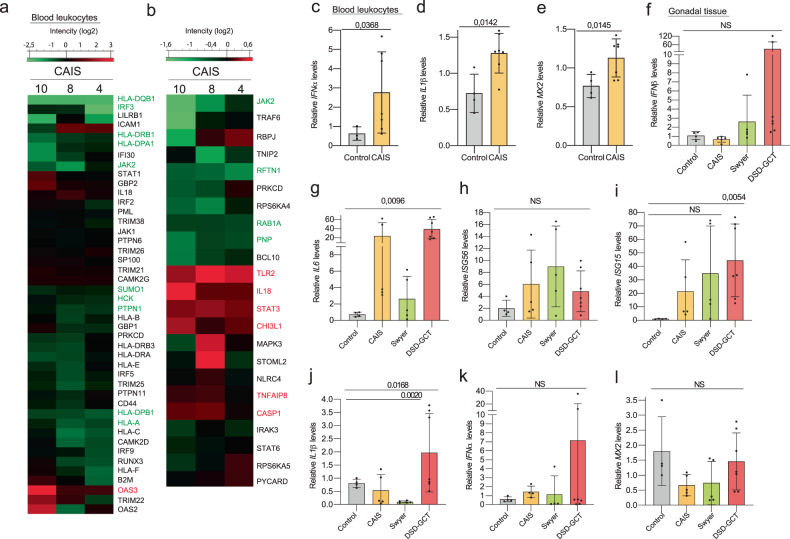
Innate immune response in blood of individuals with CAIS and dysgenic gonads. Whole proteome analysis of leukocytes from blood of individuals with CAIS with GO for interferon (**a**) and GO for interleukin (**b**). Relative gene expression levels of *IFNα* (**c**), *IL1β* (**d**), *MX2* (**e**) in blood from individuals with CAIS and *IFNβ* (**f**), *IL6* (**g**), *ISG56* (**h**), *ISG15* (**i**), *IL1β* (**j**), *IFNα* (**k**), *MX2* (**l**) in dysgenic gonads. ANOVA and unpaired t test were used for statistical analysis; p values, means and standard deviations are shown on the plots.

IFN-specific proteome analysis in leukocytes from individuals with CAIS showed a major decrease and a specific increase of IFNβ-induced OAS3 (Fig. 2a, Supplementary dataset 4). On the other hand, the ILs-specific proteome showed an overall increase in proteins, including STAT3, IL18 and TLR2 (Fig. 2b, Supplementary dataset 5). Furthermore, we found upregulated transcripts of *IFNα*, *IL1β*, and the target gene *MX2* in blood from the CAIS group (Fig. 2c-e). However, we did not observe statistically significant changes of *IFNγ*, *IL12α* and *TNF* (Extended data Fig. 1c-e), as well as some other target genes (Extended data Fig. 1f-i).

Previously, we reported upregulation of *IFNβ* and *IL6* in the blood of individuals with CAIS [14], but in gonadal tissue, only *IL6* exhibited an increase (Fig. 2f, g). *IFNβ* showed no increase, which correlated with non-statistical changes in the expression of its target genes *ISG15* and *ISG56* (Fig. 2g, h). Consistent with the results from the blood of individuals with CAIS, we also observed upregulation of *IL1β* associated with GCT, which could potentially explain the increase in the IFN-target gene *ISG15* (Fig. 2 i-k). In addition, MX2 did not exhibit any significant differences, indicating a relatively weak dependence of IFNβ in the gonads of individuals with DSD (Fig. 2l).

In summary, our analysis revealed rather stronger elevation in the IL-specific proteome and transcripts expression in the blood and gonads in the CAIS group. Conversely, the DSD-GCT group exhibited IFN simulation in both blood and gonadal tissue, accompanied by IL production in the presence of GCT.

### AR disappears with increase of DSD gonadal deterioration

**Fig. 3:**
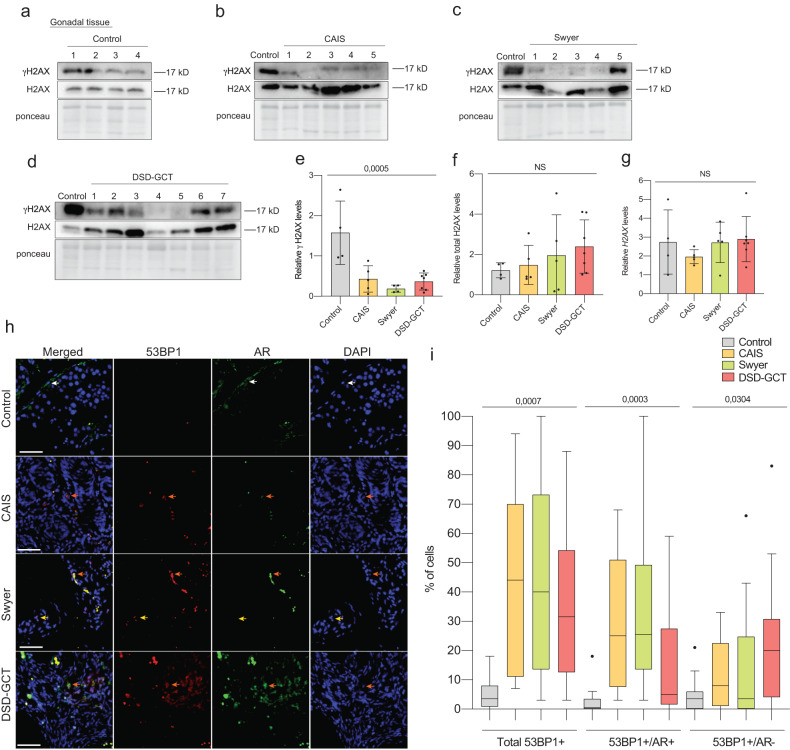
DNA damage in gonadal tissue of individuals with DSD. Immunoblot of γH2AX, total H2AX levels of gonadal tissue samples from the control (**a**), CAIS (**b**), Swyer (**c**) and DSD-GCT (**d**) groups. Quantification of γH2AX (**e**), total H2AX (**f**) protein levels relative to ponceau, serving as the loading control. **g** Relative H2AX levels in gonadal tissue samples. **h** Immunofluorescence of 53BP1 and AR in dysgenic gonads. **i** Quantification of percentages of the total 53BP1+ cells, 53BP1 + /AR+ cells and 53BP1 + /AR- cells in the control (*n* = 1340), CAIS (*n* = 5081), Swyer (*n* = 1637) and DSD-GCT (*n* = 3553) groups from (**h**). n-number of analyzed cells. ANOVA was used for statistical analysis; *p* values, means and standard deviations are shown on the plots. Scale 10 μm.

Specific phosphorylation of histone H2AX (γH2AX) serves as a marker for double-strand breakes in genomic DNA, initiating the activation of DNA damage response mechanisms [38]. In control testis, we observed stable expression of γH2AX levels, similar to total H2AX levels, which can be attributed to the ongoing meiosis during spermatogenesis (Fig. 3a). In contrast, dysgenic gonadal tissue from individuals with DSD, characterized by impaired or absent gametogenesis, exhibited significantly inhibited relative levels of γH2AX (Fig. 3b-e), while total H2AX and its transcripts remain unchanged (Fig. 3b-d, f, g).

To assess the extent of gonadal tissue deterioration, we examined DNA damage by analyzing the presence of 53BP51. We observed an increase in 53BP1+ cells in tissues from all DSD individuals (Fig. 3h, I, Extended data Fig. 2a-d, yellow arrows label 53BP1+ cells, orange arrows label 53BP1 + /AR+ cells). The number of AR+ cells was reduced in DSD-GCT gonads, likely due to tissue degeneration (Fig. 3h, Extended data Fig. 2a, b, white arrows label AR+ cells). 53BP1 + /AR+ cells exhibiting signs of DNA damage were upregulated in DSD gonads, while diminished in the DSD-GCT group (Fig. 3h, I, Extended data Fig. 2c, d). Furthermore, as the severity of dysgenic tissue degeneration increased, cells gradually lost AR and acquired 53BP1 expression.

### DNA damage response is compromised in individuals with DSD

**Fig. 4:**
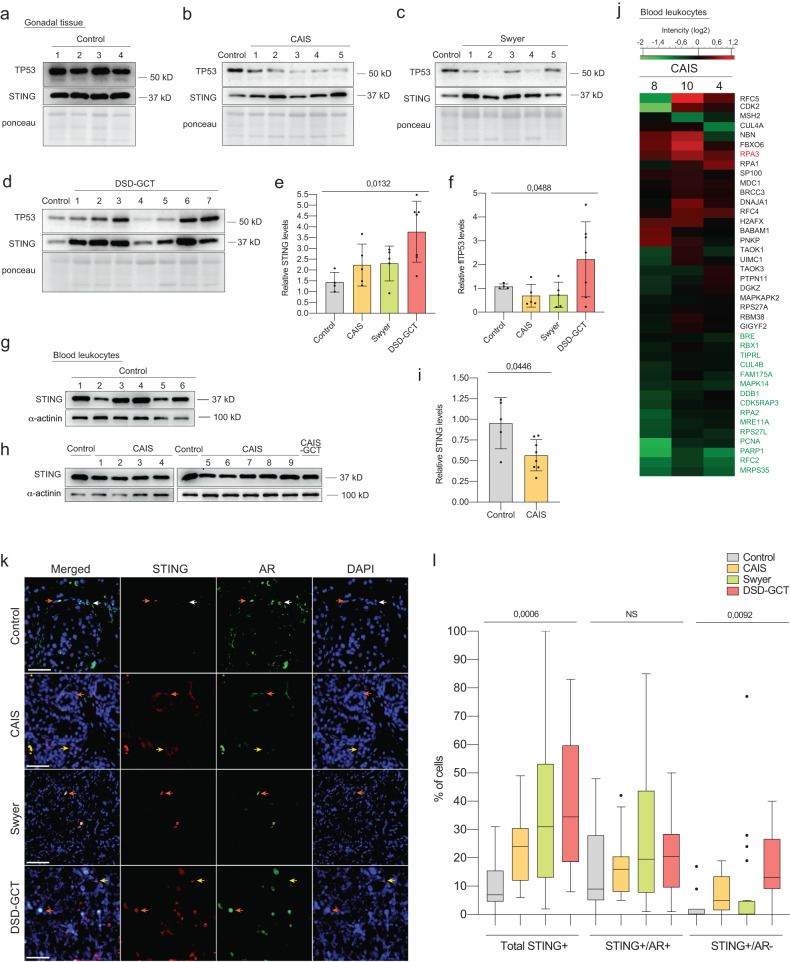
STING correlates with inhibited DDR in blood and gonadal tissue of individuals with DSD. Immunoblotting of TP53 and STING in gonadal tissue samples from control (**a**), CAIS (**b**), Swyer (**c**) and DSD-GCT (**d**) groups. Quantification of STING (**e**) and TP53 **(f**) protein levels relative to the loading control ponceau. Immunoblotting of STING in blood samples from control (**g**) and CAIS (**h**) groups. **i** Quantification of relative STING protein levels normalized to a-actinin. **j** Heat map presenting DDR-specific proteome from the whole-proteome analysis of CAIS samples. **k** Immunofluorescence of STING and AR in dysgenic gonadal tissue. White arrows label STING-/AR+ cells, orange arrows label STING + /AR+ and yellow arrows label STING + /AR- cells. **l** Quantification of percentages of total STING + , STING + /AR+ and STING + /AR- cells in control (*n* = 1347), CAIS (*n* = 2431), Swyer (*n* = 2853) and DSD-GCT (*n* = 2426) groups. n-number of analyzed cells. Unpaired t test and ANOVA were used for statistical analysis; *p* values, means and standard deviations are shown on the plots. Scale 10 μm.

STING is a critical protein involved in transmitting signals from the cytoplasm to activate innate immune response and autophagy in response to a degenerating genome [39]. We observed weak upregulation of STING in dysgenic gonads, while the GCT group exhibited a four-fold upregulation (Fig. 4a-e). Notably, TP53 protein levels were increased only in gonads of the DSD-GCT group (Fig. 4a-d, f). The mutations in common hot spot sites for protein modifications [40] could compromise DDR and potentially led to the development of GCT (Extended data Fig. 3). In a previous study, we did not observe an increase in DNA damage and TP53 levels in the blood of individuals with CAIS compared to the control [14], which is consistent with the observed decrease in STING (Fig. 4g-i). These findings were supported by a whole proteome analysis that revealed mainly decreased expression of DDR-related proteins (Fig. 4j, Supplementary dataset 6).

According to the reported increase in total STING protein levels, we only observed an increase in the number of STING + /AR- cells with increasing gonadal tissue deterioration and decreasing AR+ cells (Fig. 4k, l, Extended data Fig. 2e-g). Therefore, our findings suggest that low levels of STING may be relevant to the upregulation of innate immune response but do not support DDR activation in individuals with CAIS. Furthermore, STING appears to participate in propagation of genotoxic stress phenotypes in dysgenic gonads, especially with GCT, while AR expression is lost.

#### Genotoxic stress reflects the severity of male infertile phenotypes

**Fig. 5:**
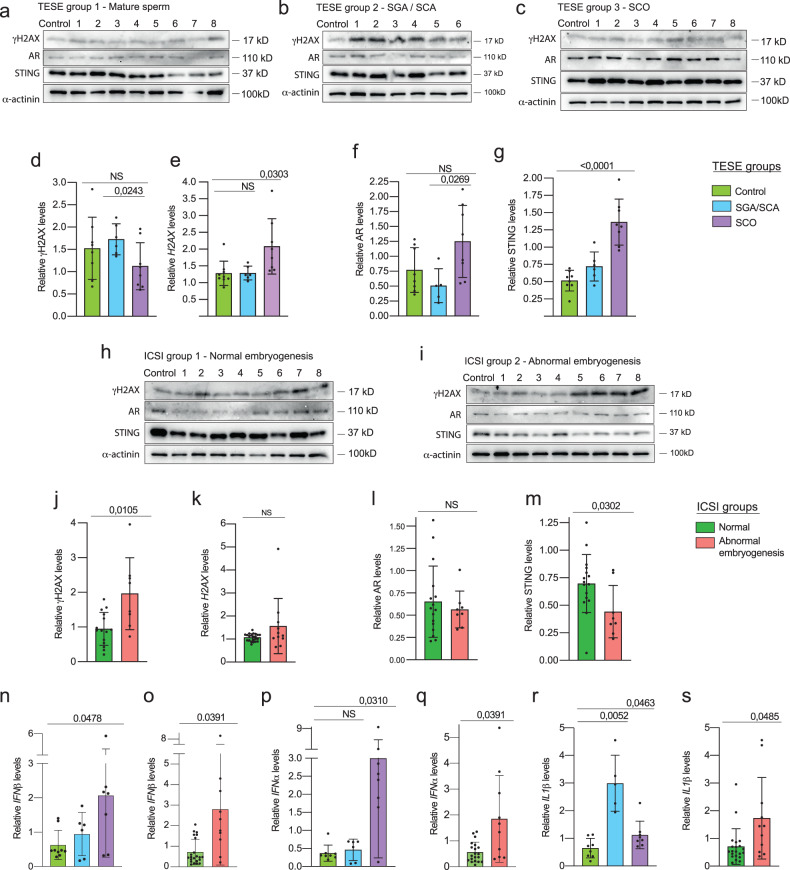
Upregulated genotoxic stress phenotypes in leukocytes of infertile men. Immunoblotting of γH2AX, AR and STING of samples from individuals of TESE groups 1 (**a**), 2 (**b**), 3 (**c**) and ICSI groups 1 (**h**) and 2 (**i**). Quantification of the blots that represent γH2AX (**d**, **j**), AR (**f**, **l**) and STING (**g**, **m**) levels normalized to α-actinin, used as the loading control. Relative levels of *H2AX* transcripts of the samples from TESE groups (**e**) and ICSI groups (**k**). Relative levels of transcripts of *IFNβ* (**n**, **o**), *IFNα* (**p**, **q**) and *ILβ1* (**r**, **s**). Unpaired t test and ANOVA were used for statistical analysis; p values, means and standard deviations are shown on the plots.

In order to illustrate the general nature of the observed above phenotypes we assessed the presence of genomic instability in infertile men, often characterized by secondary deterioration of the gonads. We examined peripheral blood samples obtained from men undergoing testicular sperm extraction (TESE) that were categorized according to the degree of testicular tissue deterioration: 1) individuals with seminiferous tubules containing mature sperm, served as the control group; 2) individuals with seminiferous tubules exhibiting arrested spermatogenesis at spermatogonia (SGA) and spermatocyte (SCA) stages, with a low probability of detecting spermatozoa; 3) individuals with SCO, where tubules solely consist of somatic cells (Supplementary Table 3). There were no changes between the control and SGA groups (Fig. 5a-d), while SCO-samples showed an elevation of *H2AX* transcripts, suggesting the presence of DNA damage phenotype in individuals with SCO [41] (Fig. 5e). We also found that AR expression was not changed in comparison to controls, while it was higher by approximately two-fold in leukocytes of SCO- compared to SGA/SCA-TESE groups (Fig. 5 a-c, f). Importantly, we observed a gradual upregulation of STING protein levels in the blood of men from TESE groups, correlating with the severity of gonadal tissue deterioration (Fig. 5a-c, g).

In order to further investigate the broader manifestation of the DNA damage phenotype, we also examined men, who had undergone the ICSI procedure (Supplementary table 4). We observed a two-fold increase in DNA damage represented by γH2AX levels in leukocytes of infertile males who had worse ICSI outcomes, which only correlated with two-fold decreased STING levels (Fig. 5h-m). We also observed a statistically significant upregulation of *IFNβ* transcripts, up to four-folds, in both the TESE and ICSI groups (Fig. 5n, o). Interestingly, *IFNα* [42] showed upregulation only in individuals with SCO and the ICSI group with abnormal embryogenesis (Fig. 5p, q). *ILβ1* was upregulated up to six-fold in the SGA/SCA and two-fold in the SCO TESE groups, as well as in the “abnormal” ICSI group compared to the control (Fig. 5r, s). Further investigation revealed that type I IFN target genes like *MX2*, *ISG15* and *ISG56* were upregulated exclusively in the SGA/SCA TESE, possibly reflecting AR inhibition in this group (Extended data Fig. 4a-c). Importantly, the ICSI group with abnormal embryogenesis did not showed a similar increase, consistent with the low STING levels described above (Extended data Fig. 4d-f).

These findings suggest that genotoxic stress phenotypes in the blood from the groups with testicular tissue deterioration are associated with deregulated AR and STING.

#### DNA damage phenotype in testicular tissue of men with NOA

**Fig. 6:**
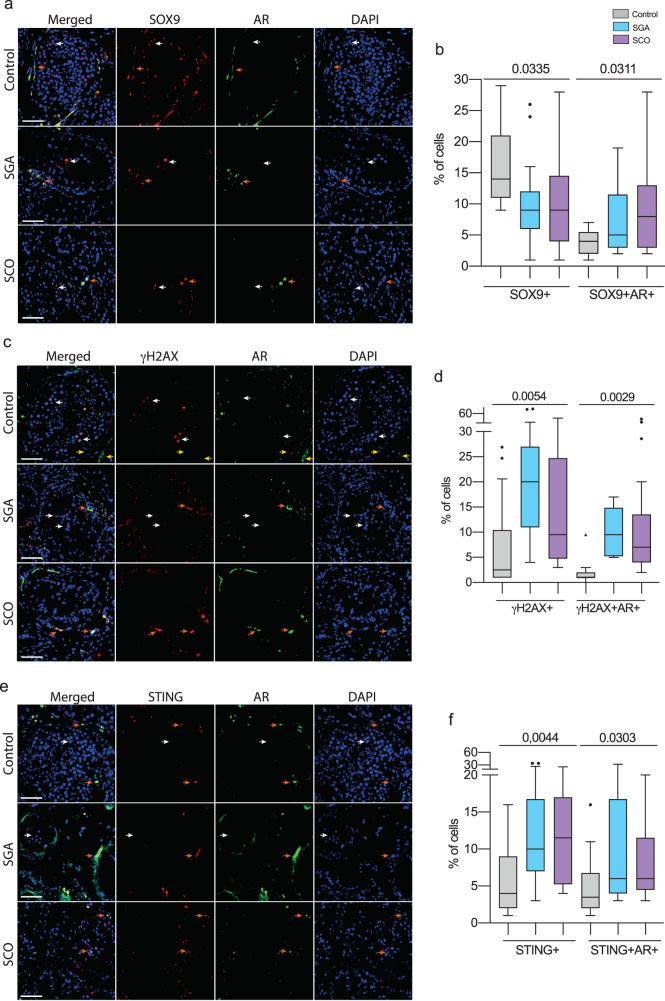
Genotoxic stress phenotypes in testicular tissue samples of individuals from TESE groups. Fluorescent double immunohistochemistry on testicular tissue of individuals from the control group and men with SGA and SCO diagnosis of AR with SOX9 (**a**). **a** White arrows indicate Sertoli cells with SOX9-positive nuclei; orange arrows point to AR-positive Sertoli cells with SOX9 in the cytoplasm. Quantification of the percentage of AR and SOX9 single and double positive cells (**b**; *n*(Control)=4994, *n*(SGA) = 7028, *n*(SCO) = 7724). Immunofluorescence of AR with γH2AX proteins (**c**). **c** white arrows indicate γH2AX-positive AR-negative spermatocytes; orange arrows point to AR/γH2AX-double positive degenerating testicular/Sertoli cells; yellow arrow indicates AR-positive γH2AX-negative cells on the periphery of the seminiferous tubules. Quantification of the percentage of AR and γH2AX single and double positive cells (**d**; *n*(Control)=4671, *n*(SGA) = 5448, *n*(SCO) = 5626). Immunofluorescence of AR with STING proteins (**e**). **e** white arrows point to STING/AR-double negative cells inside the tubules; orange arrows indicate STING/AR-double positive degenerating cells on the periphery of the tubules. Quantification of the percentage of AR and STING single and double positive cells (**f**; *n*(Control)=6899, *n*(SGA) = 4552, *n*(SCO) = 5554). ANOVA test was used for statistical analysis; *p* values, means and standard deviations are shown on the plots. Scale 10 μm. n - total number of analyzed cells.

AR was present in the cytoplasm of SOX9-positive Sertoli cells at the periphery, but was absent in the middle of the seminiferous channels in all studied groups (Fig. 6a, white arrows). The percentage of SOX9-positive cells decreased with the severity of testicular tissue degeneration, while the presence of SOX9 + /AR+ cells increased (Fig. 6a, b, orange arrows, Extended data Fig. 4g-i).

In the control group, we observed approximately 2% γH2AX-positive cells, predominantly corresponding to spermatocytes and spermatogonia cells undergoing meiosis [43] (Fig. 6c, d, white arrows, Extended data Fig. 4c, d). In testes with SGA, the percentage of γH2AX-positive cells was ten times higher, corresponding to accumulation of arrested immature spermatogonia cells localized primarily in the center of tubules. SCO TESE samples exclusively contained γH2AX + /AR+ cells, confirming the absence of normal spermatogonia formation in testes with SCO (Fig. 6c, d, orange arrows, Extended data Fig. 4j, k). Interestingly, there were AR + /γH2AX- cells primarily located at the periphery of the tubules in the control testes (Fig. 6c, d, yellow arrows).

We observed a significant increase in the percentage of STING + /AR+ cells in testicular tissues with SGA/SCO (Fig. 6e, f, orange arrows), which correlated with upregulated levels of STING in blood of individuals with SCO (Fig. 5g). Therefore, these findings highlight an intriguing correlation between genotoxic stress phenotypes in gonadal tissue and support our previous observations in blood.

### CAIS individuals are protected from the deregulation of DDR

**Fig. 7:**
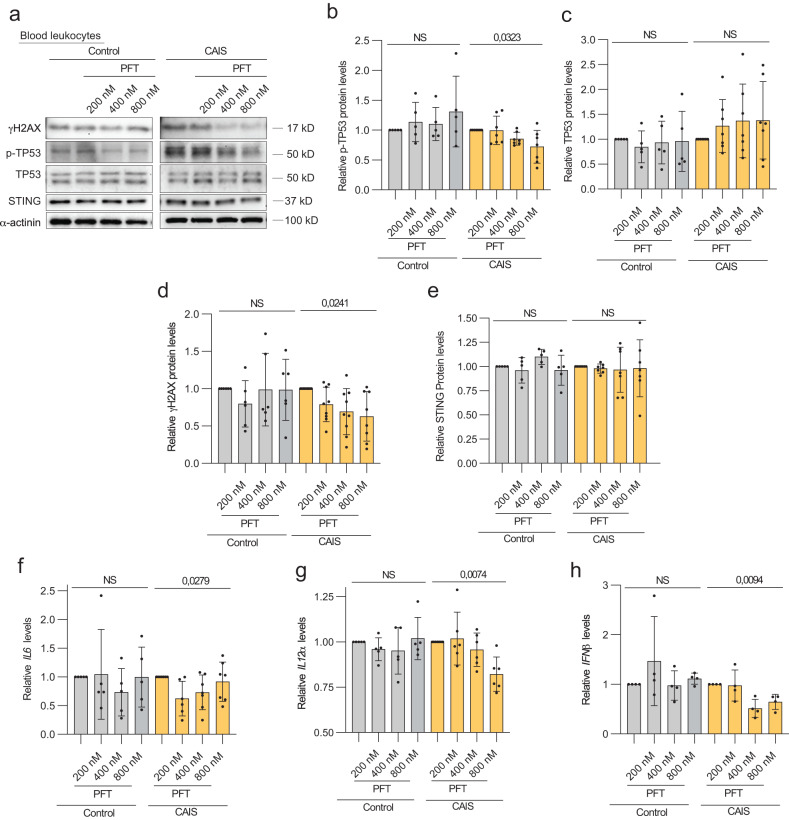
TP53 inhibition in fresh blood of individuals with CAIS in vitro. **a** Immunoblotting of γH2AX, p-TP53, TP53 and STING in the control and CAIS fresh blood samples treated with PFT. Quantification of p-TP53 (**b**), TP53 (**c**), γH2AX (**d**), STING (**e**) relative protein levels normalized to the loading control α-actinin of immunoblot (**a**). Relative gene expression levels of IL6 (**f**), IL12α (**g**) and IFNβ (**h**) in control and CAIS groups upon exposure to PFT. ANOVA test was used for statistical analysis; p values, the ratio of means of DMSO vs PFT samples and standard deviations are shown on the plots.

To determine whether there is a biological significance of the specific AR and DDR phenotypes in individuals with DSD, we conducted functional studies using peripheral blood leukocytes. Fresh blood samples were cultured in vitro in the presence of the TP53 inhibitor Pifithrin-μ (PFT). We observed p-TP53 protein inhibition confirming efficiency of the drug, while total TP53 levels remained unchanged in blood leukocytes from individuals with CAIS (Fig. 7a-c). Importantly, we observed a gradual downregulation of γH2AX levels, which was supported by the absence of upregulation of STING in the same CAIS samples (Fig. 7a, d, e). In contrast, blood leukocytes from the control group did not exhibit statistically significant changes under the same experimental conditions. We also showed a gradual decrease in the expression of *IL6*, *IL12α* and *IFNβ* upon PFT treatment specifically in the CAIS group (Fig. 7f-h). However, *IFNα*, *IFNγ, IL1β, TNF*, and the target genes *MX1* and *MX2* did not change (Extended data Fig. 5a-f). Therefore, leukocytes from individuals with CAIS exhibited relative resistance to DNA damaging conditions and higher sensitivity than the control group upon TP53 inhibition.

### Cross-talk between AR and genome DNA damage

**Fig. 8:**
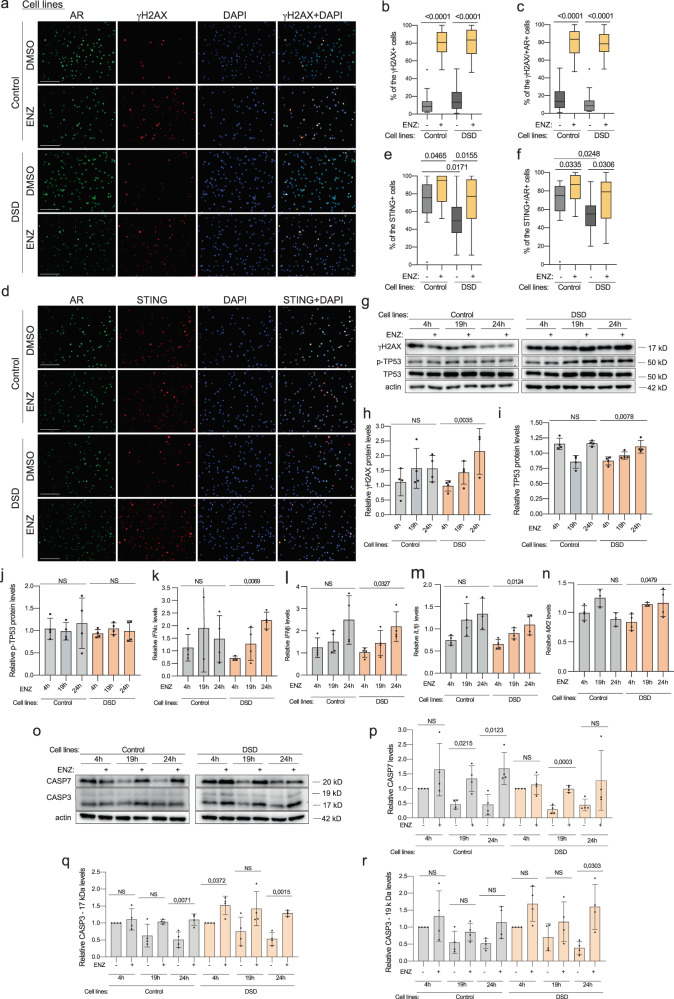
AR inhibition in lymphoblastoid cell lines from individuals with DSD. Immunofluorescence of γH2AX (**a**) or STING (**d**) and AR of lymphoblastoid cell lines from the control and DSD group treated with AR inhibitor ENZ (100 μM) for 19 h. Quantification of percentages of γH2AX+ (**b**), γH2AX + /AR+ (**c**), STING+ (**e**), STING + /AR+ (**f**) cells from immunostaining (**a**, **d**). Four different control and DSD cell lines were used and at least 1200 cells were calculated per condition. Immunoblotting for γH2AX, p-TP53, TP53 (**g**) or CASP7, CASP3 (**o**) of the cell lines samples of the control and DSD groups treated with ENZ (100 μM). Quantification of γH2AX (**h**), TP53 (**i**), p-TP53 (**j**), CASP7 (**p**), CASP3-17kD (**q**), CASP3-19kD (**r**) relative levels normalized to actin, used as the loading control of the immunoblotting (**g**, **o**). Relative gene expression levels of *IFNα* (**k**), *IFNβ* (**l**), *IL1β* (**m**), *MX2* (**n**). ANOVA and unpaired t test were used for statistical analysis; p values, the ratio of means of DMSO vs ENZ samples and standard deviations are shown on the plots.

To investigate whether long-term AR inhibition can interact with the innate immune response in the context of sustained genomic instability, we utilized lymphoblastoid cell lines derived from peripheral blood leukocytes of individuals with DSD, exhibiting DNA damage phenotypes [14]. These cell lines were treated with androgen inhibitor Enzalutamide (ENZ) for up to 1 day. Consistent with previous findings in other cell types [21], we observed an increase in the number of γH2AX+ cells, also co-positive for AR following ENZ treatment in both control and DSD-cell lines (Fig. 8a-c, Extended data Fig. 5g-i). These observations were accompanied by an increase in the number of STING+ and STING + /AR+ cells, particularly in the DSD group, following incubation with ENZ (Fig. 8d-f, Extended data Fig. 5j, k). At the protein level, we primarily observed a significant upregulation of γH2AX specifically in DSD-samples (Fig. 8g, h), which was accompanied by an upregulation of TP53, but not p-TP53- protein levels (Fig. 8g, i, j). *IFNα, IFNβ, IL1β* and *MX2* were upregulated in DSD cell lines compared to control cell lines (Fig. 8k-n).

ZIP9, recently discovered AR, has been proposed as an alternative target for ENZ [44]. We have not detected ZIP9 presence in lymphoblastoid cell lines, therefore it’s irrelevance to the observed phenotypes upon ENZ treatment (Extended data Fig. 6a). However, ZIP9 should be taken into account when androgen signaling is studied in the leukocyte isolated from the fresh blood (Extended data Fig. 6b).

Interestingly, together with the DNA damage phenotype, lymphoblastoid cell lines promoted apoptosis via upregulation of Caspases (CASP) upon ENZ treatment (Fig. 8o-r). Both cell lines exhibited upregulation of CASP7 (Fig. 6o, p), but CASP3 17 kD and 19 kD isoforms showed a stronger response in the DSD group (Fig. 8o, q, r). These data demonstrated strong genotoxic stress phenotypes of cells from individuals with DSD and their greater susceptivity to apoptosis stimulation upon AR inhibition in the context of sustained genomic instability.

## Discussion

In this study, we have demonstrated the crucial role of androgens in regulating genome integrity within the context of the innate immune response. To investigate this, we examined various groups of infertile individuals. CAIS is a common cause of DSD that exhibited significantly upregulated expression of proteins associated with innate immune response in the blood. Similar deregulation of innate immune response, independent of inflammation, has been implicated in DDR, suggesting a potential link between genome integrity and innate immune response [35–37]. Interestingly, the single protein in AR-relevant proteome, MED30, was found to be upregulated that has been also reported to have connections to DDR [45]. Therefore, we have provided evidence for the upregulation of specific genes associated with the innate immune response in the blood of all infertile men. This upregulation involves both IFN and IL pathways, although the absence of upregulation of any IFN target genes suggests that the SCO group may be more susceptible to IL-dependent regulation. Actually, all myeloid progenitors express AR and can exhibit a direct inhibition of type 2 immune responses by androgens [20]. For instance, AR inhibition restores the production of the specific cell types, such as group 2 innate lymphoid cells (ILC2s) and the relevant IL33 signaling-driven inflammation in the lungs of mice [46]. This mechanism may potentially be relevant to IL-specific phenotypes in SCO group. While we did not observe changes in *TNF* expression in the blood of individuals with CAIS, it does not preclude the involvement of apolipoprotein E (APOE)-relevant mechanisms in azoospermia and other DSD-relevant phenotypes, as it was reported in microglia [47].

Furthermore, decreased STING was specific for the ICSI group with abnormal embryogenesis, distinguishing it from other study groups characterized by more severe infertility phenotypes. STING is a crucial component of the cGAS-STING pathways, responsible for transmitting signals from cytoplasmic genomic DNA to initiate the innate immune response [14, 39]. In our current study, we contribute to the understanding of interaction between AR, STING and the innate immune response induced by DNA damage.

The increased proportion of AR-positive cells observed in the tissue from SGA and SCO groups likely indicates a reduction of cells at various stages of spermatogenesis. Furthermore, the immunofluorescence data suggest that AR is expressed in poor quality Sertoli cells, which are likely nonfunctional and located at the periphery of the seminiferous tubules. The presence of these cells becomes more prominent as testicular tissue degeneration worsens. We observed an elevated presence of degenerating AR+ cells exhibiting DNA damage at the periphery of seminiferous tubules in testicular tissues with SGA and SCO. These findings, along with the identification of STING in AR+ cells, indicate the presence of genomic instability as a contributing factor to the infertility phenotypes observed in these individuals. The increase of AR+ cells displaying DNA damage and expressing STING is a characteristic feature of deteriorating gonadal tissue in individuals with DSD and NOA.

DNA damage repair is predominantly mediated by homologous recombination (HR)-proficient cells, whereas HR-deficient cells exhibit limited repair capacity. This is based on the variability in alterations of DDR between germinal and somatic cells, observed in cancer patients [48]. The DDR status plays a critical role in androgen deprivation therapy for prostate cancer. In line with this, our in vitro functional study demonstrated variable cell responses. Firstly, ENZ treatment in the presence of genomic instability led to apoptosis in cells with compromised DDR, potentially selecting for cells that are resilient to AR inhibition and capable of regulating DNA damage phenotypes through functional DDR. We observed that upregulation of the innate immune response in DSD-cell lines correlated with increased DNA damage and TP53-dependent DDR following ENZ treatment. Secondly, cells from individuals with CAIS exhibited protection from TP53 inhibition, resulting in decreased DNA damage likely due to their unique DDR status caused by constant AR inhibition.

The negative impact of genotoxic agents on male fertility, as well as changes in DDR-specific gene expression in men with azoospermia, has been known for some time [49]. However, the underlying molecular mechanisms have remained unclear. Our research reveals that androgen signaling can be concurrently inhibited to counteract the genotoxic phenotypes that can be mitigated with drugs such as enoxacin or autophagy inhibitors, thereby positively influencing spermatogenesis [14, 50]. This insight potentially shed light on the fundamental causes of infertility of men with azoospermia. Focusing on this common phenotype may offer a reasonable solution to approach male infertility, especially when specific gene mutations are not identified. If extended in vitro tests yield positive results, targeted chemical therapy can be considered for individuals with azoospermia. Furthermore, the better understanding of the common mechanisms connecting molecular phenotypes in peripheral blood with gonadal pathology will facilitate early diagnosis of infertility and its potential causes. However, considering limitations of in vitro studies such as unbalanced nutrient supply and oxygen exchange, risk of DNA mutagenesis and cell contamination, these results should be applied with great caution to patients’ studies. Preferably, these findings should be validated through consequent testing on 3D in vitro and animal in vivo models.

In principle, any disease associated with genotoxic stress phenotypes may share similar underlying mechanisms, which allows us to maintain a broad outlook on our findings and their translational relevance. Genomic instability stands as a primary factor in the development of cancer, as well as neurodegenerative conditions such as Alzheimer’s disease (AD) and Parkinson’s disease (PD) [51, 52], as well as Fanconi anemia (FA) [53], and general senescence phenotypes [54]. Genotoxic stress, often linked to aneuploidy, is characteristic of conditions like trisomy of chromosome 21 in Down syndrome (DS), which we have recently associated with increased innate immune response and deregulated autophagy [18, 19, 39]. It is conceivable that androgen function may be altered in individuals diagnosed with these diseases, given their frequent infertility. Indeed, studies have shown the potential benefits of therapy influencing activity of androgen signaling in AD and FA [55, 56]. Notably, the therapeutical suppression of innate immunity has coincidentally improved fertility in patients with AD and PD [57].

Guidelines for providing holistic, multidisciplinary care from childhood have long been proposed for individuals with DSD [58]. However, suitable specialized care for adolescents is still not consistently accessible, which could significantly enhance their quality of life. A better understanding of the molecular mechanisms responsible for gonadal dysgenesis in individuals with DSD is essential for identifying new preventive treatments and early diagnostic approaches of GCT. For example, timely intervention to reduce genotoxic phenotypes such as DNA damage levels, inflammation, and imbalanced autophagy may promote germ cells specification and decrease the risk of GCT when administered early enough. This has the potential to enable fertility preservation and increase the feasibility of artificial reproduction treatments, which should be considered as a viable option and weighted against the risks of GCT. Consequently, reducing the need for prophylactic gonadectomy in clinical practice.

In conclusion, our study has demonstrated an interconnected relationship between the status of spermatogenesis and the expression of markers in peripheral blood. The gene expression profiles of innate immune response genes, as well as the levels of STING and AR in the blood of infertile men, can serve as predictive indicators of tissue deterioration and the likelihood of successful sperm retrieval. This knowledge can provide a rational for TESE in patients with NOA. Importantly, these findings also contribute to the development of preventive treatments for individuals with CAIS. The decision to retain dysgenic gonads should be based on an assessment of the defined risks associated with GCT development, which is connected to gonadal tissue deterioration and, as we demonstrated, specific gene expression patterns in peripheral blood. This understanding of the mechanisms and causes of tumorigenesis provides valuable information for predictive diagnosis of GCT, allowing to keep the gonads and the potential preservation of fertility in individuals with androgen insensitivity [59].

## Material and methods

### Patients’ material

Individuals with Differences of Sex Development (DSD) were enrolled into study during routine regular checkups in the Department of Gynecological Endocrinology and Fertility Disorders. The study was approved by the ethical committee and the samples were collected with written patients’ consent. For this study we analyzed 23 individuals with DSD. DSD- individuals included individuals with Swyer and Complete Androgen Insensitivity (CAIS) Syndromes. The study groups are listed with additional information (Supplementary table 1 and 2). The sample underwent standard characterization procedures in genetics and histology facility at Heidelberg University Hospital. Control group contain samples from fertile men and women.

Infertile men with NOA were enrolled into the study during routine diagnostic procedures in the Department of Gynecological Endocrinology and Fertility Disorders of Heidelberg University Hospital. The testicular tissue samples for TESE from these individuals were procured by the Urology Department. In case men underwent ICSI, the information on fertilization and embryo development was provided by the IVF laboratory of our department. For this study we analyzed samples from total 51 infertile men (22 from TESE groups, 29 from ICSI groups). There was no female infertility factor reported for these cases. The TESE group with the “motile sperm: was used as the control for other TESE groups with the defective spermatogenesis. The individuals participating in the study are listed with additional information (Supplementary tables 3, 4). The project was approved by the ethical committee, and the samples were collected with written patient’s consent. We didn’t observe any statistically significant age differences among individuals within these groups.

We collected data on fertilization rates and embryo development at Days 2 and 5 (Supplementary table 4). Based on the success ratios, we divided the samples into two groups: “normal” (average success ratio >50%) and “abnormal” (average success ratio ≤50%) embryo development. The average developmental rates were calculated based on the percentages of fertilized oocytes and normal development of day 2 (good quality 4-cell embryo) and day 5 (good quality, at least full blastocyst) embryo development. Since some of the couples underwent embryo transfer on day 2 and, therefore, we also calculated average developmental rates based only on fertilisation and developmental day 2, which did not alter the content of the patients’ groups. The “normal” group served as the control for comparison with the group exhibiting “abnormal” embryo development. There were no statistically significant age differences between individuals within these ICSI groups, neither between men and women, nor between the women from these two different groups.

### Medically Assisted Reproduction

Controlled ovarian stimulation was performed to obtain oocytes. After denudation metaphase II oocytes were used for TESE-ICSI. The presence of two pronuclei was scored as normal fertilization. Embryos were cultured individually in droplets under oil up to day 5 in an incubator at 37° C, 5% O_2_ and CO_2_ adjusted to reach a pH of 7.2 to 7.4. Cleavage stage embryos were assessed for cell number, degree of fragmentation and cell symmetry according to clinical standard operative procedures (SOP). Blastocysts were scored as it was previously described [60]. All embryos were derived from normally fertilized oocytes (2PN) and criteria for good morphology and developmental rate are the following: G1 for the cleavage/compaction stages and blastulation with an A and B score for ICM and TE, according to Alpha Scientists (2011) [61]. No embryos were produced or used for clinical research, only information obtained from clinical records was collected. Data on the fertilization and embryo development after ICSI with the sperm from infertile men is represented in the Supplementary table 4.

### Leukocytes isolation from the peripheral blood

Blood was collected in EDTA and subjected to leukocytes isolation according to the stablished protocol. 10 ml of blood was mixed with 30 ml of lysis buffer (155 mM NH_4_Cl, 10 mM KHCO3, 0,1 mM EDTA ph 7.4) and incubated 30’ on ice. Then the mix was centrifuged for 10’ at 1200 rpm at 4°C. The pellet was washed three times with 10 ml lysis buffer and then subjected for DNA, RNA and protein isolation with NucleoSpin® TriPrep kit (Marcherey-Nagel).

### EBV-transformed lymphoblastoid cells

EBV stock was prepared from cultures of EBV-transformed marmoset cells, strain B95-8. The B95-8’ strain of EBV was used to transform human B lymphocytes as previously described [62]. 10 ml EDTA-blood was mixed with 30 ml of Lysis buffer (155 mM NH_4_Cl, 10 mM KHCO3, 0,1 mM EDTA ph 7.4) and left for 30’ on ice, then centrifuged for 10’ at 1200 rpm and 4°C. The pellet was washed three times with 10 ml of Lysis buffer and resuspended with 3 ml of culture medium, containing RPMI Medium 1640 (with stable L-Glutamin) Cat.# 61870010, ThermoFisher Scientific) plus 10% FBS (Cat.# 10500064, ThermoFisher Scientific), penicillin (50 U/ml), streptomycin (25 pg/ml) (Cat.# 15140122, ThermoFisher Scientific), gentamycin (Cat.# 15710, ThermoFisher Scientific), NEA (Cat.# 11140035, ThermoFisher Scientific) and sodium pyruvate (Cat.# 11360070, ThermoFisher Scientific). After virus stock was added at 1 /10 final dilution to a cell suspension containing 2 X l0 ^6^ cells/ml. The virus was allowed to adsorb to the cells for 2 hours at 37°C. Cells were then pelleted by low-speed centrifugation and resuspended at 2 ×10^6^ cells/ml in culture medium.

The cell lines of four DSD-individuals (three Turner and one Swyer syndrome) and controls (two males and two females) were cultured with 100 μM Enzalutamide (ENZ, MDV3100, cat.# S1250, Selleckhem) for 4, 19 and 24 hours, DMSO was used as a control [63].

### Blood culture with drugs

For each condition we prepared 10 cm dishes with 10 ml culture medium, containing 2 ml of blood, collected in citrate and containing approximately 5×10^6 leukocytes per 1 ml; 15% FCS (Cat. #10500064, Life Technologies); 20 μl PHA-L (Cat.# 00-4977-03, Life Technologies). We added 200 nM, 400 nM or 800 nM pifithrin-μ (PFT, cat.# SC-45050, Santa Cruz) [64]. DMSO was used as the control. This short-term culture of primary patient cells aimed to minimize the influence of artificial conditions. After 1 h incubation at 37°C, 5%CO2 cells were collected, washed 2 times with 1xPBS (Cat. # 14190-094, Gibco) and subjected to leukocytes isolation procedure as described above. DNA, RNA and protein isolation from the leukocytes was performed with NucleoSpin® TriPrep kit (Marcherey-Nagel). The experiment was executed at least four times.

### Mass spectrometry

Proteome-wide expression profiling of leukocytes from three individuals with CAIS (Supplementary table 1, samples 4, 8, 10) three fertile controls (Supplementary table 2, samples 1, 2, 8), was supported by the Core Facility for Mass Spectrometry & Proteomics (CFMP) at the Center for Molecular Biology at University Heidelberg (ZMBH). 50 µg of protein was precipitated using Wessel-Flügge method [65] and protein pellet was dissolved in 8 M Urea buffer. After reduction and alkylation for 30’ at RT using 10 mM TCEP and 40 mM CAA, Lys-C was added at 1:100 enzyme:protein ratio and incubated for 4 h at 37° C. Mixture was diluted 1:4 with 50 mM TEAB pH 8.5 and trypsin was added in ratio 1:50. After overnight incubation at 37° C, samples were acidified, desalted on self-made C18 Empore® extraction discs (3 M) StageTips [66], concentrated in a SpeedVac and stored at -20° C until measured.

Samples were suspended in 0.1% TFA and an equivalent to 1 µg of peptides was analyzed using Ultimate 3000 liquid chromatography system coupled to an Orbitrap QE HF (Thermo Fisher). An in-house packed analytical column (75 µm x 200 mm, 1.9 µm ReprosilPur-AQ 120 C18 material Dr. Maisch, Germany) was used. Mobile phase solutions were prepared as follows, solvent A: 0.1% formic acid / 1% acetonitrile, solvent B: 0.1% formic acid, 89.9% acetonitrile.

Peptides were separated in a 120 min linear gradient started from 3% B and increased to 23% B over 100 min and to 38% B over 20 min, followed by washout with 95% B. The mass spectrometer was operated in data-dependent acquisition mode, automatically switching between MS and MS2. MS spectra (m/z 400–1600) were acquired in the Orbitrap at 60,000 (m/z 400) resolution and MS2 spectra were generated for up to 15 precursors with normalized collision energy of 27 and isolation width of 1.4 m/z.

The MS/MS spectra were searched against the Swiss-Prot Homo sapiens protein database (UP000005640, June 2020, 20531 sequences) and a customized contaminant database (part of MaxQuant, MPI Martinsried) using Proteome Discoverer 2.5 with Sequest HT (Thermo Fisher Scientific). A fragment ion mass tolerance was set to 0.02 Da and a parent ion mass tolerance to 10 ppm. Trypsin was specified as enzyme. Carbamidomethyl was set as fixed modification of cysteine and oxidation (methionine), acetylation (protein N-terminus) and methionine loss (protein N-terminus) as variable modifications. Peptide quantification was done using precursor ion quantifier node with Top N Average method set for protein abundance calculation and N for Top N set to 3.

For comparison of each CAIS group with the control we calculated the protein levels fold change ratios. The same datasets were used to analyze innate immune response, inflammation, AR-signaling, interferon and leukocyte, and DDR-specific proteome. Presence of at least one unique peptide is required for identification of reported protein groups. Minimum of two ratio counts were required for the quantitation based only on unique and razor peptides. Statistically significant differential expression of proteins was defined by T-test, adjusted for multiple testing (FDR = 0.05, S0 = 0.1, Perseus) and used for log2 intensities of samples from DSD-groups, normalized to control (Supplementary datasets 1-6).

The proteomics data used in this manuscript are deposited in the ProteomeXchange Consortium (http://proteomecentral.proteomexchange.org) via the PRIDE partner repository and can be accessed with a code PXD033635.

### Sequencing

We used genomic DNA of individuals with DSD for sequencing. We analyzed exons 5-9 and TP53 gene according to the established protocol (IARC, 2019). The primers used for exon 5 F- ttcaactctgtctccttcct; R-cagccctgtcgtctctccag; exon 6 F-gcctctgattcctcactgat; R- ttaacccctcctcccagaga; exon 7 F-cttgccacaggtctccccaa; R- aggggtcagaggcaagcaga; exon 8-9 F-ttgggagtagatggagcct; R-agtgttagactggaaacttt. The sequencing was performed using BigDye Terminator v1.1 Cycle Sequencing Kit (Applied Biosystems, Cat. no. 4337450) and the ABI PRISM 3100 Genetic Analyzer. Sequencing Data that support the findings of this study have been deposited into open access NCBI GeneBank with the accession codes OR247844 - OR247855. Sequence results from forward and reverse primers were considered for the evaluation of real variants to avoid technical errors. DNA alignments to reference TP53 sequence are presented (Supplementary data set 7).

### Western blotting

Protein from leukocytes was analyzed via Western Blot analysis following modified protocol [67]. The protein concentration was determined by Protein Quantification Assay (Cat.# 740967,Macherey-Nagel). 10 µg of the protein was separated by SDS-PAGE electrophoresis, then transferred onto PVDF membrane (Immobilon-P membrane (Cat.# IPVH00010, Millipore). The membrane was blocked with 5% skim milk (Cat. # T145, Carl Roth) or 3% BSA (Cat. # 8076.4, Carl Roth) 1 h at RT. Then incubated at 4°C overnight with primary antibodies. After washing three times with buffer TNT (50 mM Tris, 150 mM NaCl, 5 mM EDTA and 0.05% Tween 20; pH 7.6), the membrane was incubated with corresponding secondary antibodies (peroxidase conjugated Goat Anti-Rabbit IgG (Cat.# 111-035-046, Dianova) or peroxidase conjugated Goat Anti-Mouse IgG (Cat.# 115-035-062, Dianova) for 1 h at RT. Protein bands were visualized by enhanced chemiluminescence with an ECL kit (SuperSignal West Femto Substrat (Cat. # 34095, Thermo Scientific). Image development was performed on iBright FL1000 Imager (Cat.# A32748, Invitrogen). Primary antibodies used: γH2AX (ab11174, Abcam; 1 mg/ml; 1/5000), H2AX (ab124781, Abcam, 1/5000), actin (SC1616, Santa Cruz, 1/10000), α-actinin (SC-17829, Santa Cruz Biotechnology; 200 µg/ml; 1/30.000), AR (M3562, DAKO; 1/300), STING (13647 S, Cell Signaling; 1/5000), TP53 (M7001, Agilent, DAKO, 1/500), p-TP53 (Cell Signaling, CS9281, 1/300), CASP7 (CS9491, Cell signaling, 1/1000), CASP3 (CS9664, Cell signaling, 1/800), ZIP9 (ab137205, Abcam, 1/300). Every point in the plots of data for WB analysis represents one individual patient sample or experimental measurement, total sample size is represented by 3-10 points on the graph. The means of each measurement/experiment were calculated and plotted for presentation and for statistical analysis using GraphPad. Two-sided unpaired t test was used, means and p-values, s.d. and error bars are shown on the graphs. Original immunoblotting files are presented (Supplementary data file).

### Quantitative real-time PCR

1 μg RNA was used for cDNA synthesis with Superscript II (Cat. # 18064022, Invitrogen). qPCR analysis was performed on an ABI Prism 7900HT Fast Real-Time PCR System (Applied Biosystems). The cycling conditions were as follows: 50°C for 2 min, denaturation at 95°C for 10 min followed by 40 cycles at 95°C for 15 s, and a combined annealing and extension step at 60°C for 60 s. The TaqMan assays (Termofisher) used: *H2AX* (Hs00266783_s1), *IFNβ* (Hs01077958_s1)*, ISG15* (Hs00192713_m1)*, ISG56* (Hs0175197_m1)*, IFNα* (Hs00855471_g1)*, ILβ1* (Hs01555410_m1)*, MX2* (Hs01550814_m1)*, HPRT* (Hs99999909_m1), *TBP* (Hs00427620_m1), *MX1* (Hs00895608_m1), *IFNγ* (Hs00989291_m1), *IL6* (HS00174131_m1), *IL12α* (Hs01073447_m1), *TNF* (Hs00174128_m1), IFI16, (Hs00986757_m1), *IFITM2* (Hs00829485_sH). Every point in the plots of data for qRT-PCR analysis represents one individual patient sample or experimental measurement, total sample size is represented by 3-10 points on the graph. The means of each measurement/experiment were calculated and plotted for presentation and for statistical analysis using GraphPad. Two-sided unpaired t test was used, means and p-values, s.d. and error bars are shown on the graphs. No RT control was used.

### Immunofluorescence

Gonadal tissue sections were provided by the tissue bank of the National Centre for Tumor Diseases (NCT, Heidelberg, Germany) in accordance with the regulations of the tissue bank and the approval of the ethics committee of Heidelberg University. Immunostaining was performed on 4 μm dehydrated FFPE tissue sections. Antigen retrieval was performed by immersing the slides in 10 mM citrate buffer (pH 6.0) in a steamer for 10 min. Next, tissue sections were allowed to cool and then kept at room temperature for 20 min. Slides were washed twice in PBS and three times in washing buffer (PBS containing 0,1% Triton X-100). Tissue sections were incubated in 3% H_2_O_2_/Methanol for 10 min. Sections were washed three times with washing buffer and then blocked with blocking buffer (5% goat serum (cat. # S-1000, Vector) in washing buffer) for 1 h at room temperature. Tissue sections were incubated with primary antibody in a humid box at 4 °C for 16 h. The primary antibody were diluted in 1% goat serum/washing buffer. After three washes in washing buffer secondary fluorescence-conjugated antibodies (Goat Anti-Rabbit Alexa Fluor 488, cat. # A32723, Invitrogen, Goat Anti-Mouse Alexa Fluor 594, cat.# A11037, Invitrogen) were diluted to a concentration of 2 µg/ml in 1% goat serum/washing buffer and incubated for 1 h at room temperature in a dark humid box. Slides were then rinsed in washing buffer five times for 3 min each and nuclei were stained with 4′,6-diamidino-2-phenylindole (DAPI, cat # D-1388, Sigma) at a concentration of 1 μg/ml for 10 min at RT. Sections were then rinsed three times with water for 3 min each before being mounted and coverslips were applied using ProLong Glass Antifade Mountant (cat. #P36980, Invitrogen).

For Immunostaining of non-adherent lymphoblastoid cells glas cover slips were coated with Poly-D-Lysin (cat.# A3890401, Gibco) according to the manufactuer´s manual. Glas cover slip (12 mm Ø) was placed onto the bottom of a well in a 24-well culture plate. Lymphocytes were centrifugated into the pellet in a conical tube at 250 x g for 5 minutes, the supernatant was aspirated and the pellet was resuspended in PBS in the concentration of approximately 3 ×10^5^ cells/ ml. 0,3 ml of the cell suspension were transferred to a cover slip in a well and allowed to stay for 30-60 min at room temperature [68]. After cells adhered, the culture plate was washed with PBS and then fixed for 10’ at room temperature with 4% Paraformaldedye/PBS. Fixed cells were washed two times with PBS, each for 5’ before being permeabilized with 0,5% Triton x-100 for 10’. Cells are washed once again with PBS for 5’ and then blocked with blocking buffer (5% goat serum ;cat. # S-1000, Vector; in PBS) for 1 h at room temperature. The blocking solution is replaced with primary antibodies in a humid box at 4 °C for 16 h. The primary antibodies were diluted in 1% goat serum/PBS. Cells were subsequently washed three times with PBS for 5’ and incubated with secondary fluorescence-conjugated antibodies. Secondary antibodies were diluted to a concentracion of 2 µg/ml in 1% goat serum/PBS and incubated for 1 h at room temperature in a dark humid box. Cells are washed three times with PBS for 5’ and nuclei are counterstained with DAPI (cat # D-1388, Sigma) at a concentration of 1 μg/ml for 10’ at room temperature. Cells were then rinsed five times with water for 3 min each. A drop of ProLong Glass Antifade Mountant (cat.#P36980, Invitrogen) is added to the center of a microscope slide and the coverslip was gently transferred cell-side down onto this surface after being gently lifted from the well bottom.

The imaging was performed using a fluorescence microscope (LEICA, DMLB), using a HQ camera SpectraCube (Applied Spectral Imaging, Mannheim, Germany) and a 20x-air or 63x-oil objective (PL FLUOTAR 20x/0,5 PH2, HCX PL APO 63x/1,32-0,6 OIL, PL FLUOTAR 100x/1,3 OIL PH3) under the control of the Spectral imaging acquisition software 2.6 (Applied Spectral Imaging, Germany). For immunofluorescent analysis, 1000-5000 s of cells were analyzed using automatic software ImageJ. The means of each measurement/experiment were calculated and plotted for presentation and for statistical analysis using GraphPad. Two-sided unpaired t test was used, means and p-values, s.d. and error bars are shown on the graphs. Primary antibodies used anti-γ-H2AX rabbit polyclonal antibody (0,25 μg/mL, cat. #ab11174, Abcam), anti-STING (D2P2F) rabbit monoclonal (1/50 dilution, 13647 S, Cell Signaling Technology), anti-AR mouse monoclonal (M3562, DAKO; 1/75) and anti-SOX9 rabbit polyclonal (2 μg/ml, SC-20095, Santa Cruz Biotechnology), anti-53BP1 rabbit monoclonal (ab175933 Abcam, 1/1000, 0,275 mg/ml).

### Supplementary information

Supplementary tables 1-4

Extended data figure 1-6

original data

checklist

Supplementary dataset 1

Supplementary dataset 2

Supplementary dataset 3

Supplementary dataset 4

Supplementary dataset 5

Supplementary dataset 6

Supplementary dataset 7

## Data Availability

Data are available on request from the contact person.
