# Supplementary material for: Low androgen signaling rescues genome integrity with innate immune response by reducing fertility in humans
Source: Cell Death Dis. 2024 Jan 11;15(1):30. doi: 10.1038/s41419-023-06397-5 (PMC10784536; doi:10.1038/s41419-023-06397-5)
Supplement: Supplementary file 1 — Supplementary tables 1-4 [file 41419_2023_6397_MOESM1_ESM.docx]

**Supplementary table 1.** Samples of leukocyte from peripheral blood of individuals with DSD and the controls.

|  | DSD groups | Age | Karyotype | Phenotype | Malignant Tumor  (gonadal biopsy) | Gonadal tissue histology |
| --- | --- | --- | --- | --- | --- | --- |
|  | CAIS |  |  |  |  |  |
| 1 | 1 | 9 | 46, XY | Female | Not found |  |
| 2 | 2 | 17 | 46, XY | Female | Not found |  |
| 3 | 3 | 18 | 46, XY | Female | Not found | hyperplastic Leydig cells |
| 4 | 4 | 17 | 46, XY | Female | Not found |  |
| 5 | 5 | \| 32 \| \| --- \| | 46, XY | Female | Not found | Differentiated Sertoli-Leydig cells tumor, secondary Sertoli cells hyperplasia, no germ cells tumor, no malignancy OCT4+, TSPY+, DDX3Y+ |
| 6 | 6 | 20 | 46, XY | Female | Dysgerminoma | OCT4+, SALL4+, D2-40+, Ki67+, but βHCG-, AFP-. |
| 7 | 7 | 29 | 46, XY | Female | Not found | Negative for AFP, OCT4, SALL4, PLAP, S100, CD117, beta-HCG |
| 8 | 8 | 45 | 46, XY | Female | Not found | Negative for S100, SALL4, AFP, C2-40, OCT4, PLAP, beta-HCG |
| 9 | 9 | 14 | 46, XY | Female | Not found |  |
| 10 | 10 | 18 | 46, XY | Female | Not found |  |
| 11 | 11 | 38 | 46, XY | Female | Not found |  |
|  | Swyer |  |  |  |  |  |
| 12 | 1 | 24 | 46, XY | Female | Not found |  |
| 13 | 2 | 16 | 46, XY | Female | Not found |  |
| 14 | 3 | 12 | 46, XY | Female | Not found |  |
| 15 | 4 | 10 | 46, XY | Female | Not found |  |
| 16 | 5 | 18 | 46, XY | Female | Not found |  |
| 17 | 6 | 16 | 46, XY | Female | Not found |  |
| 18 | 7 | 17 | 46, XY | Female | Not found |  |
|  | Swyer-GCT |  |  |  |  |  |
| 19 | 1 | 16 | 46, XY | Female | Dysgerminoma | CD117+, PLAP+ |
| 20 | 2 | 18 | 46, XY | Female | Seminoma/  Dysgerminoma | PAS+ |
| 21 | 3 | 17 | 46, XY | Female | Dysgerminoma | CD117+, D2-40+, OCT4+, PLAP+, SALL4+ |
| 22 | 4 | 32 | 46, XY | Female | Dysgerminoma, Gonadoblastoma | AFP+, β-HCG+ |
| 23 | 5 | 6 | 46, XY | Female | Dysgerminoma | KiA10+, AP + |
|  | Controls |  |  |  |  |  |
| 24 | 1 | 28 | 46, XY | Male |  |  |
| 25 | 2 | 30 | 46, XY | Male |  |  |
| 26 | 3 | 25 | 46, XY | Male |  |  |
| 27 | 4 | 28 | 46, XY | Male |  |  |
| 28 | 5 | 26 | 46, XY | Male |  |  |
| 29 | 6 | 28 | 46, XY | Male |  |  |
| 30 | 7 | 35 | 46, XY | Male |  |  |
| 31 | 8 | 26 | 46, XX | Female |  |  |
| 32 | 9 | 28 | 46, XX | Female |  |  |
| 33 | 10 | 27 | 46, XX | Female |  |  |
| 34 | 11 | 25 | 46, XX | Female |  |  |
| 35 | 12 | 38 | 46, XX | Female |  |  |

|  | DSD groups | Age | Karyotype | Phenotype | Malignant Tumor  (gonadal biopsy) | Gonadal tissue, histology info |
| --- | --- | --- | --- | --- | --- | --- |
|  | CAIS |  |  |  |  |  |
| 9 | 1 | 14 | 46, XY | Female | Not found | Testis-like gonad, right side |
| 10 | 2 | 18 | 46, XY | Female | Not found | Testis-like gonad, right side |
| 4 | 3 | 17 | 46, XY | Female | Not found | Testis-like gonad, left side |
| 2 | 4 | 17 | 46, XY | Female | Not found | Testis-like gonad, right side |
| 7 | 5 | 29 | 46, XY | Female | Not found | Testis-like gonad, right side. Negative for AFP, OCT4, SALL4, PLAP, S100, CD117, beta-HCG |
|  | Swyer |  |  |  |  |  |
| 12 | 1 | 18 | 46, XY | Female | Not found | Strand gonad, left side |
|  | 2 | 24 | 46, XY | Female | Not found | Strand gonad, right side |
| 16 | 3 | 17 | 46, XY | Female | Not found | Strand gonad, right side |
| 17 | 4 | 16 | 46, XY | Female | Not found | Strand gonad, left side |
| 18 | 5 | 16 | 46, XY | Female | Not found | Strand gonad, right side |
|  | Swyer-GCT |  |  |  |  |  |
|  | 1 | 16 | 46, XY | Female | Gonadoblastoma | Left gonad. |
| 19 | 2 | 16 | 46, XY | Female | Dysgerminoma | Strand gonad, right side. CD117+, PLAP+ |
| 20 | 3 | 18 | 46, XY | Female | Dysgerminoma | Left gonad. PAS+ |
| 21 | 4 | 20 | 46, XY | Female | Gonadoblastoma | Strand gonad, right side* |
|  | 5 | 20 | 46, XY | Female | Dysgerminoma | Strand gonad, left side |
| 22 | 6 | 17 | 46, XY | Female | Gonadoblastoma | Right gonad** |
| 23 | 7 | 17 | 46, XY | Female | Dysgerminoma | Left gonad. CD117+, D2-40+, OCT4+, PLAP+, SALL4+ |
|  | Controls |  |  |  |  |  |
| 24 | 1 | 57 | 46, XY | Male | Not found | Normal testis |
| 25 | 2 | 51 | 46, XY | Male | Not found | Normal testis |
| 26 | 3 | 7 | 46, XY | Male | Not found | Normal testis |
| 27 | 4 | 38 | 46, XY | Male | Not found | Normal testis |

**Supplementary table 2.** Gonadal tissue samples of individuals with DSD and the controls.

*Samples 4 and 5 are from the same individual.

** Samples 6 and 7 are from the same individual.

| Study groups | | Age |
| --- | --- | --- |
| TESE group 1,  motile sperm | |  |
| 1 | 1 | 37 |
| 2 | 2 | 35 |
| 3 | 3 | 43 |
| 4 | 4 | 48 |
| 5 | 5 | 29 |
| 6 | 6 | 45 |
| 7 | 7 | 30 |
| 8 | 8 | 31 |
| Average | | 37,25±7,3 |
| TESE group 2,  SGA, SCA | |  |
| 9 | 1 | 34 |
| 10 | 2 | 31 |
| 11 | 3 | 37 |
| 12 | 4 | 43 |
| 13 | 5 | 36 |
| 14 | 6 | 41 |
| Average | | 37±4,4 |
| TESE group 3,  SCO | |  |
| 15 | 1 | 36 |
| 16 | 2 | 32 |
| 17 | 3 | 44 |
| 18 | 4 | 45 |
| 19 | 5 | 32 |
| 20 | 6 | 35 |
| 21 | 7 | 36 |
| 22 | 8 | 32 |
| Average | | 36,5±5,23 |

**Supplementary table 3.** Infertile men in TESE groups.

**Supplementary table 4.** ICSI patients’ groups.

| ICSI groups | | Age | | ICSI outcome | | | | | | | |
| --- | --- | --- | --- | --- | --- | --- | --- | --- | --- | --- | --- |
|  |  |  |  | Fertilisation | | Day 2 embryo | | Day 5 embryo | | Average developmental rates, % | |
|  |  |  |  | % | N | % | N * | % | N* |  |  |
| 1 - normal development | | men | women |  |  |  |  |  |  | >50% | |
|  |  |  |  |  |  |  |  |  |  | ** | *** |
| 1 | 1 | 45 | 42 | 83 | 12 | 100 | 3 | NA | NA | NA | 91,5 |
| 2 | 2 | 41 | 32 | 55 | 9 | 100 | 2 | NA | NA | NA | 77,5 |
| 3 | 3 | 39 | 33 | 100 | 5 | 100 | 3 | 100 | 3 | 100 | 100 |
| 4 | 4 | 33 | 30 | 94 | 18 | 100 | 4 | 50 | 4 | 81,3 | 97 |
| 5 | 5 | 43 | 39 | 80 | 10 | 100 | 4 | 25 | 4 | 68,3 | 90 |
| 6 | 6 | 30 | 28 | 100 | 9 | 100 | 4 | 75 | 4 | 91,7 | 100 |
| 7 | 7 | 29 | 27 | 88 | 17 | 60 | 5 | 80 | 5 | 76 | 74 |
| 8 | 8 | 55 | 41 | 63 | 8 | 60 | 5 | 40 | 5 | 54,3 | 61,5 |
| 9 | 9 | 34 | 29 | 53 | 15 | 100 | 4 | 100 | 4 | 84,3 | 76,5 |
| 10 | 10 | 32 | 33 | 67 | 6 | 100 | 4 | 75 | 4 | 80,7 | 83,5 |
| 11 | 11 | 38 | 31 | 63 | 8 | 80 | 5 | 40 | 5 | 61 | 71,5 |
| 12 | 12 | 43 | 30 | 79 | 14 | 66 | 3 | 67 | 3 | 70,7 | 72,5 |
| 13 | 13 | 39 | 30 | 50 | 22 | 80 | 5 | 40 | 5 | 56,7 | 65 |
| 14 | 14 | 40 | 37 | 100 | 15 | 50 | 4 | 25 | 4 | 58,3 | 75 |
| 15 | 15 | 30 | 28 | 64 | 11 | 75 | 4 | 50 | 4 | 63 | 69,5 |
| 16 | 16 | 35 | 29 | 80 | 5 | 50 | 4 | NA | NA | NA | 65 |
| 17 | 17 | 43 | NA | 80 | 10 | NA | NA | NA | NA | NA | 80 |
| 18 | 18 | 30 | 31 | 70 | 10 | 75 | 4 | NA | NA | NA | 60 |
| 19 | 19 | 31 | 23 | 87,5 | 8 | 50 | 4 | NA | NA | NA | 69 |
| Average | | 37,7±6,7 | 31,8±5 |  |  |  |  |  |  |  |  |
| Sum | |  |  |  | 212 |  | 71 |  | 54 |  |  |
| 2 - abnormal development | |  |  |  |  |  |  |  |  | ≤50% | |
|  |  |  |  |  |  |  |  |  |  | ** | *** |
| 20 | 1 | 37 | 40 | 40 | 5 | NA | NA | NA | NA | NA | 33,8 |
| 21 | 2 | 36 | 33 | 14,2 | 14 | 7,14 | 14 | NA | NA | NA | 10,8 |
| 22 | 3 | 36 | 35 | 28,6 | 21 | 33,3 | 3 | NA | NA | NA | 20,6 |
| 23 | 4 | 32 | 35 | 50 | 8 | 50 | 4 | NA | NA | NA | 50 |
| 24 | 5 | 38 | 35 | 26 | 23 | 75 | 4 | 50 | 4 | 50,3 | 50,5 |
| 25 | 6 | 33 | 32 | 64 | 17 | 20 | 5 | 67 | 3 | 50,3 | 42 |
| 26 | 7 | 34 | 34 | 20 | 5 | 75 | 4 | 50 | 4 | 48,3 | 47,5 |
| 27 | 8 | 30 | 28 | 23 | 16 | 0 | 3 | NA | NA | NA | 11,5 |
| 28 | 9 | 37 | 42 | 33 | 3 | 0 | 3 | NA | NA | NA | 16,5 |
| 29 | 10 | 32 | 32 | 20 | 15 | 66,7 | 3 | NA | NA | NA | 43,35 |
| Average | | 34,5±2,5 | 34,1 ±4,16 |  |  |  |  |  |  |  |  |
| Sum | |  |  |  | 139 |  | 46 |  | 14 |  |  |

N – is the total number of the fertilised oocyte or Day 2 and Day 5embryos analysed.

% - is the percentage of fertilised oocyte or good quality embryos on Day 2 or Day 5 normalized to N.

* A decrease of the embryo number in comparison to the number of fertilized oocytes is represented due to a cryopreservation a part of the embryos.

** Average developmental rates are calculated as an average from percentages of fertilization, and Day 2 and Day 5 development success rates.

*** Average developmental rates are calculated as an average from percentages of fertilization, and Day 2 development success rates.
