# Supplementary material for: Low androgen signaling rescues genome integrity with innate immune response by reducing fertility in humans
Source: Cell Death Dis. 2024 Jan 11;15(1):30. doi: 10.1038/s41419-023-06397-5 (PMC10784536; doi:10.1038/s41419-023-06397-5)
Supplement: Supplementary file 2 — Extended data figure 1-6 [file 41419_2023_6397_MOESM2_ESM.pdf]

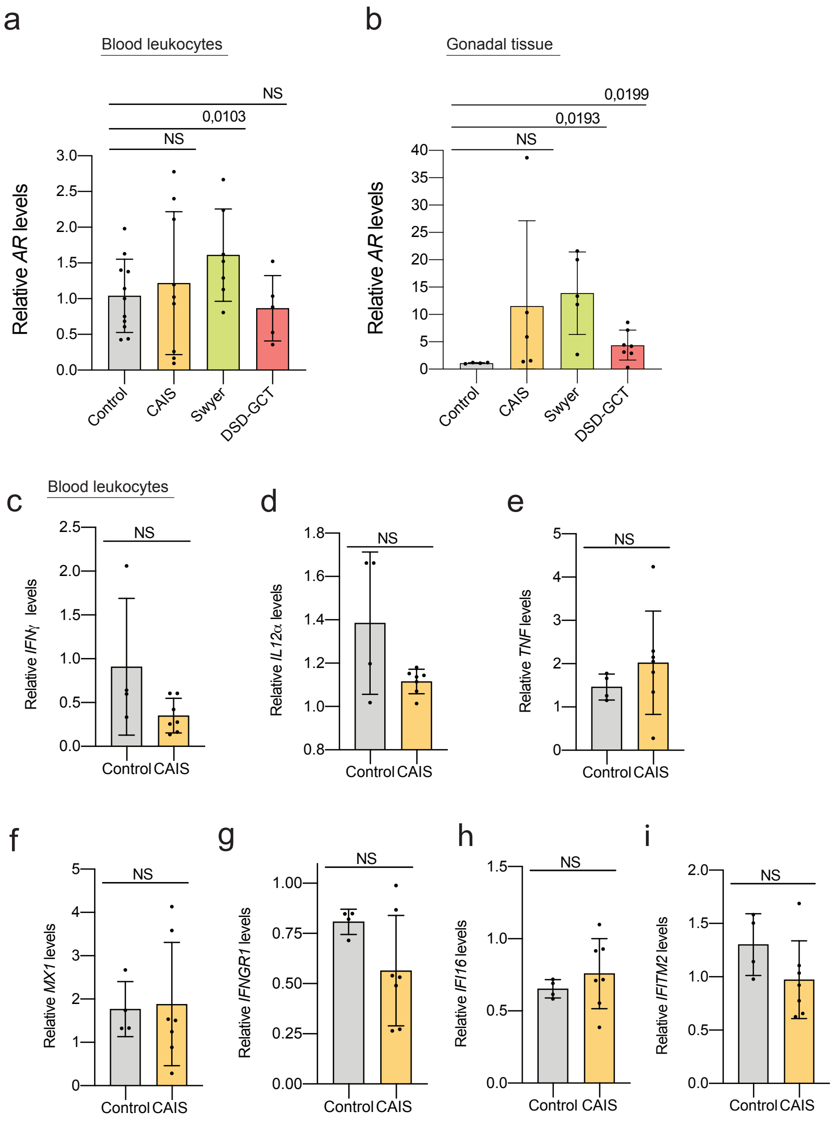

Extended data figure 1. qPCR data of *AR* expression in leukocytes from blood (a) and gonadal tissue (b) of individuals with DSD. Relative RNA levels of *IFNγ, IL12α, TNF, MX1, IFNGR1, IFI16, IRTM2* in blood of individuals with CAIS.

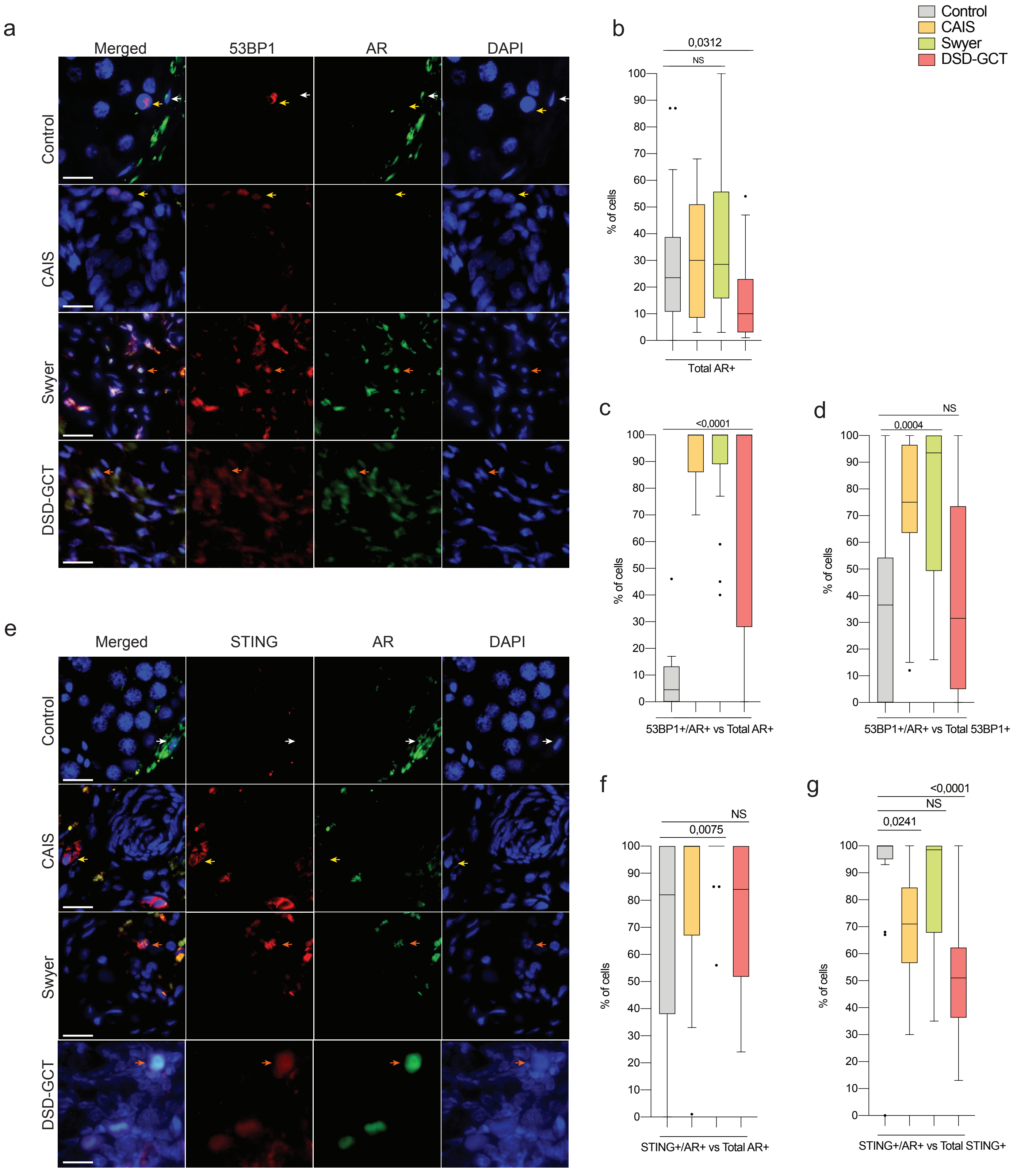

Extended data figure 2. Immunofluorescence of 53BP1 and AR (a) or STING and AR (e) in the gonads of individuals with DSD with magnification 60X. White arrows label AR+ (TP53- or STING-) cells. Yellow arrows label 53BP1+ (a) or STING+ (c) and AR- cells. Orange arrows label AR+ and 53BP1+ (a) or STING+ (c) cells. b) Percentage of AR+ cells calculated from (a) and (c). Quantification of 53+/AR+ cells vs total number of AR+ (c) or 53BP1+ (d) cells. Quantification of STING+/AR+ cells vs total AR+ (f) or STING+ (g) cells. ANOVA test was used for statistical analysis; p values, means and standard deviations are shown on the plots. Scale 3 μm.

a

```
TP53 protein control sequence
      1 meepqsdpsv epplsqetfs dlwkllpenn vlsplpsqam ddlmlspddi eqwftedpgp
     61 deaprmpeaa ppvapapaap tpaapapaps wplsssvpsq ktyqgsygfr lgflhsgtak
    121 svtctyspal nkmfcqlakt cpvqlwvdst pppgtrvram aiykqsqhmt evvrrcphhe
    181 rcsdsdglap pqhlirvegn lrveylddrn tfrhsvvvpy eppevgsdct tihynymcns
    241 scmggmnrrp iltiitleds sgnllgrnsf evrvcacpgr drrteeenlr kkgephhelp
    301 pgstkralpn ntssspqpkk kpldgeyftl qirgrerfem frelnealel kdaqagkepg
    361 gsrahsshlk skkgqstsrh kklmfktegp dsd
```

DNA-binding domain 109➔288
Common mutations
Protein modification sites
Observed alterations in DSD

b

Exon 5

```
GCT-2_gonad        YYPALNKMFCQLAKTCPVRLWVDSTPPPGTR   31
                   Y PALNKMFCQLAKTCPV LWVDSTPPPGTR
TP53_ctrl_exon5    YSPALNKMFCQLAKTCPVQLWVDSTPPPGTR   156

GCT-3_gonad        VSKMFCRTGQRLPCASVGRRQKPPPGTRGRARA   39
                   ++KMFC+   +   P    +      PPPGTR RA A
TP53_ctrl_exon5    LNKMFCQLAKTCP-VQLWVDSTPPPGTRVRAMA   161

GCT-6_gonad        QLAKDCPVNMWE*YTTPPPGTRDPHQGAIFNQSQ   46
                   QLAK CPV +W    +TPPPGTR      AI+ QSQ
TP53_ctrl_exon5    QLAKTCPVQLWV-DSTPPPGTR-VRAMAIYKQSQ   167
```

Exon 6

```
GCT-2_gonad        GLAPPQHLIRVEGNLRVEYLDDRNTFRHSVVCP   42
                   GLAPPQHLIRVEGNLRVEYLDDRNTFRHSVV P
TP53_ctrl_exon6    GLAPPQHLIRVEGNLRVEYLDDRNTFRHSVVVP   219

GCT-3_gonad        GPPQHLIRVEGNLRVEYLDDRNTFRH   83
                    PPQHLIRVEGNLRVEYLDDRNTFRH
TP53_ctrl_exon6    APPQHLIRVEGNLRVEYLDDRNTFRH   214

GCT-6_gonad        AHPQHLTRGERKFACGVFGMTGKHNFDIVWWCPYEPP   41
                   A PQHL R E       V  +  ++ F       PYEPP
TP53_ctrl_exon6    APPQHLIRVEGNLR--VEYLDDRNTFRHSVVVPYEPP   223
```

Exon 7

```
GCT-2_gonad        VGSESTTIHYNYMCNSSCMGGMNRRPILTIITLEDS   48
                   VGS+ TTIHYNYMCNSSCMGGMNRRPILTIITLEDS
TP53_ctrl_exon7    VGSDCTTIHYNYMCNSSCMGGMNRRPILTIITLEDS   260

GCT-3_gonad        VGSDCTTIHYNYMCNSSCMGGMNRRPILTIITLEDS   49
                   VGSDCTTIHYNYMCNSSCMGGMNRRPILTIITLEDS
TP53_ctrl_exon7    VGSDCTTIHYNYMCNSSCMGGMNRRPILTIITLEDS   260

GCT-6_gonad        VGSDCTTIHYNYMCNSSCMGGMNRRPILTIITLEDS   49
                   VGSDCTTIHYNYMCNSSCMGGMNRRPILTIITLEDS
TP53_ctrl_exon7    VGSDCTTIHYNYMCNSSCMGGMNRRPILTIITLEDS   260
```

Exon 8-9

```
GCT-2_gonad        ALPNNTSSSPQPKKKPLDGEYFTLQV   133
                   ALPNNTSSSPQPKKKPLDGEYFTLQ+
TP53_ctrl_exon8-9  ALPNNTSSSPQPKKKPLDGEYFTLQI   332

GCT-3_gonad        ALPNNTSSSPQPKKKPLDGEYFTLQV   133
                   ALPNNTSSSPQPKKKPLDGEYFTLQ+
TP53_ctrl_exon8-9  ALPNNTSSSPQPKKKPLDGEYFTLQI   332

GCT-6_gonad        ALPNNTSSSPQPKKKPLDGEYFTLQV   132
                   ALPNNTSSSPQPKKKPLDGEYFTLQ+
TP53_ctrl_exon8-9  ALPNNTSSSPQPKKKPLDGEYFTLQI   332
```

Extended data figure 3. Sequence of TP53 gene in gonadal tissue with GCT.
To further investigate the potential role of TP53 in STING-dependent innate immune response upregulation in the gonads of the DSD-GCT group, we sequenced the DNA binding domain to identify any hot spot mutations. We found mutations in exon 5 (samples 3 and 6) and exon 6 (sample 6) when analyzing DSD-GCT samples 2, 3 and 6.

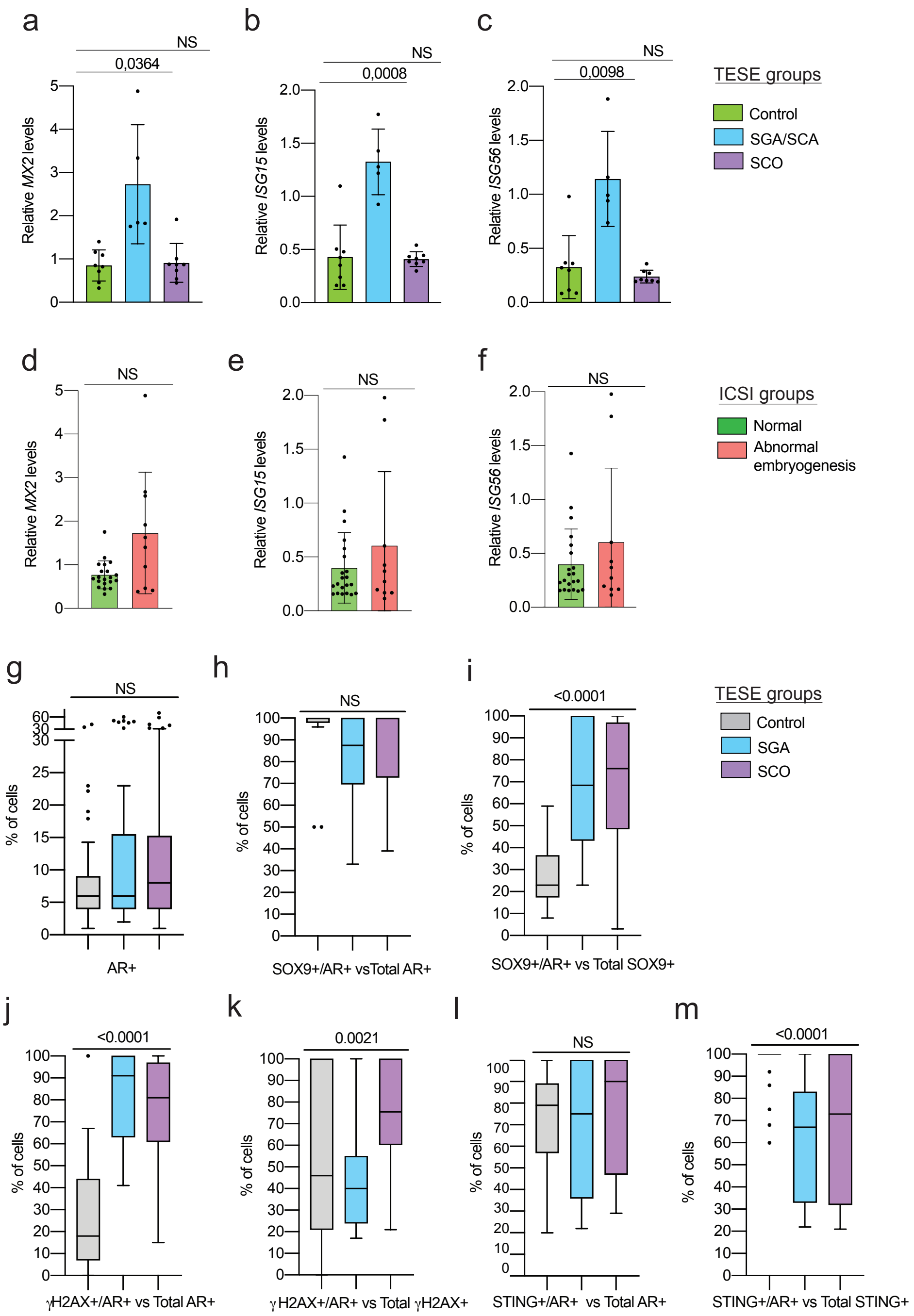

Extended data figure 4. DNA damage phenotypes in blood and gonadal tissue of men with NOA.
Relative RNA levels of *MX2* (a, b), *ISG15* (c, d) and *ISG56* (e, f) in leukocytes from periferal blood. g) Percentage of AR positive cells in the gonads. h) All AR+ cells were positive for SOX9. i) Only about 20% of SOX9-postive Sertoli cells expressed AR in the control testis, and SGA or SCO-testis exclusively contained SOX9+/AR+ cells. j) The percentage of γH2AX+/AR+ degenerating cells was also increased in tubules with SGA/SCO, which included all AR+ cells. k) Only 40% of γH2AX+ cells were AR+ in men with SGA, corresponding to degenerating portion of Sertoli cells. SCO TESE samples exclusively contained γH2AX+/AR+ cell. l, m) In tubules with SCO, all AR-positive cells were also positive for STING.

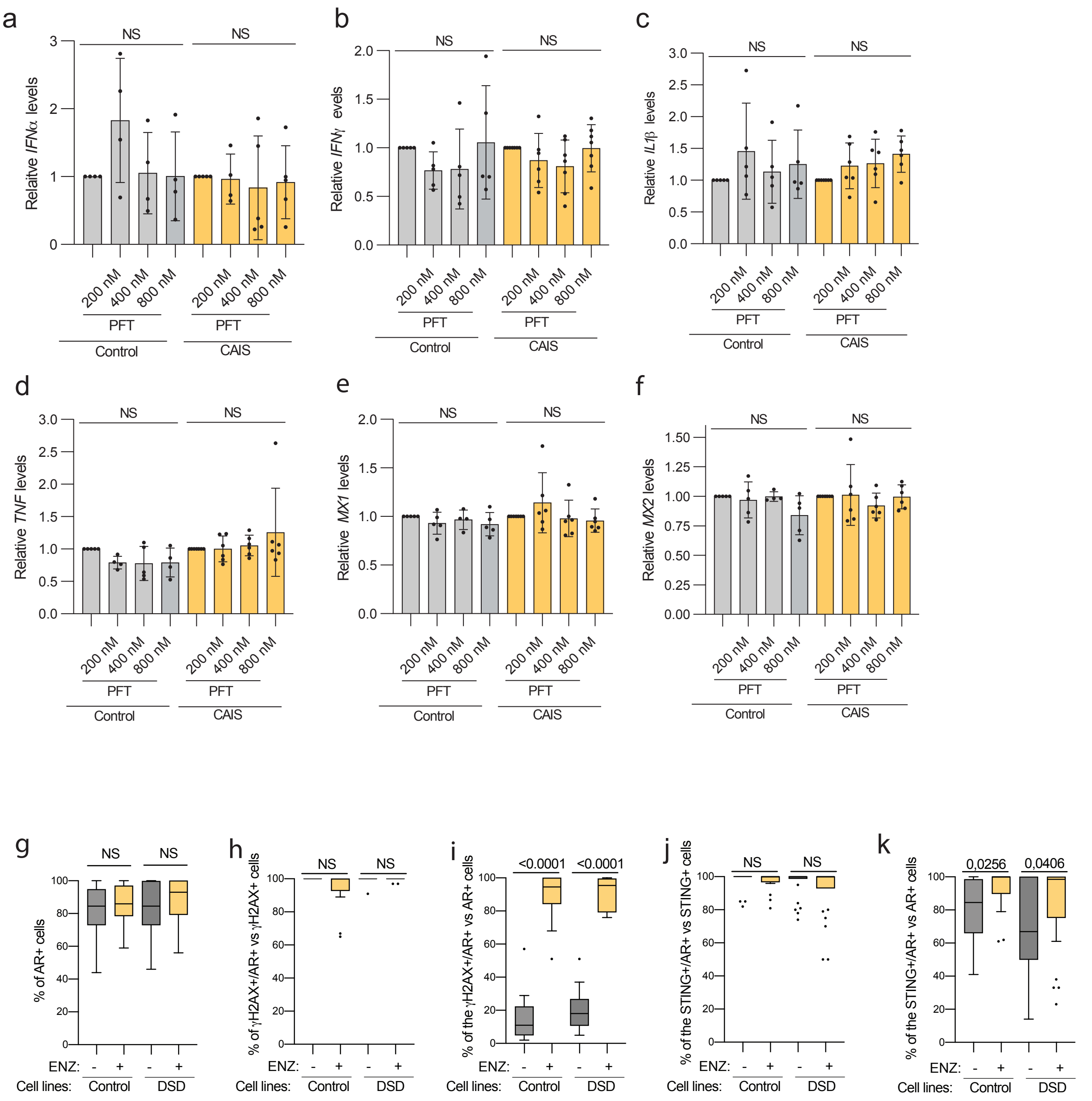

Extended data figure 5. Drugs treatment of blood from individuals with DSD.

TP53 inhibition by PFT does not affect expression of *IFN*α (a), *IFN*γ (b), *IL1*β (c), *TNF* (d), *MX1* (e), *MX2* (f). g) There were no changes in AR expression upon ENZ treatment, indicating that all γH2AX+ cells remained positive for AR as well (h). i) Significant proportion of AR+ cells did not exhibit signs of DNA damage in both DSD and control cell lines before treatment. j) All STING+ cells were co-positive for AR, and k) all AR+ cells became co-positive with STING after ENZ drug treatment.

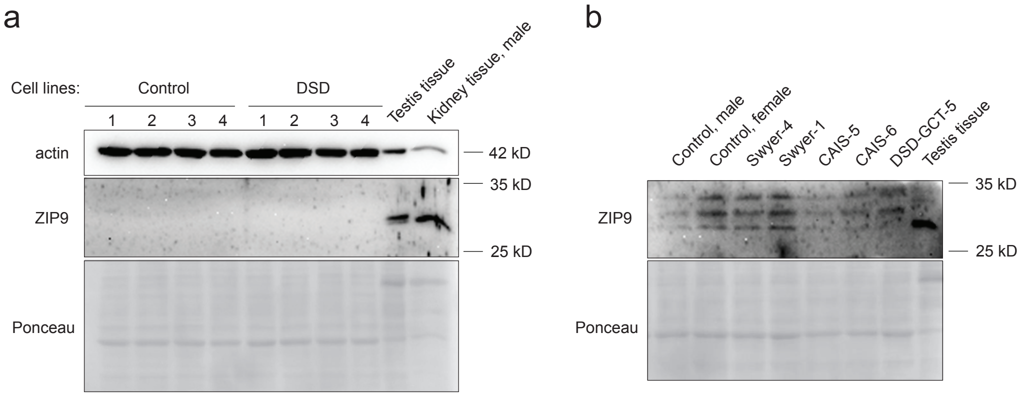

Extended data figure 6. ZIP9 expression.
ZIP9 expression was analysed with Western blot in lymphblastoid cell lines (a) and leukocytes isolated from the fresh blood (b) of individuals from control and DSD-group. Testis and kidney tissue was used as the positie controls. Actin and ponceau were analyzed as the loading controls.
