# Supplementary material for: Low androgen signaling rescues genome integrity with innate immune response by reducing fertility in humans
Source: Cell Death Dis. 2024 Jan 11;15(1):30. doi: 10.1038/s41419-023-06397-5 (PMC10784536; doi:10.1038/s41419-023-06397-5)
Supplement: Supplementary file 3 — original data [file 41419_2023_6397_MOESM3_ESM.pdf]

**Original WB data.** Some of the membranes were cut and the pieces were processed separately with different antibodies. This allowed us to analyze different markers from the same gel and also save the material.

**Figure 1d.**

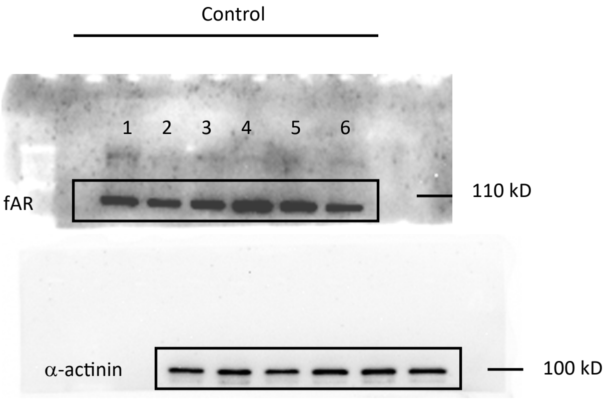


**Figure 1e.**

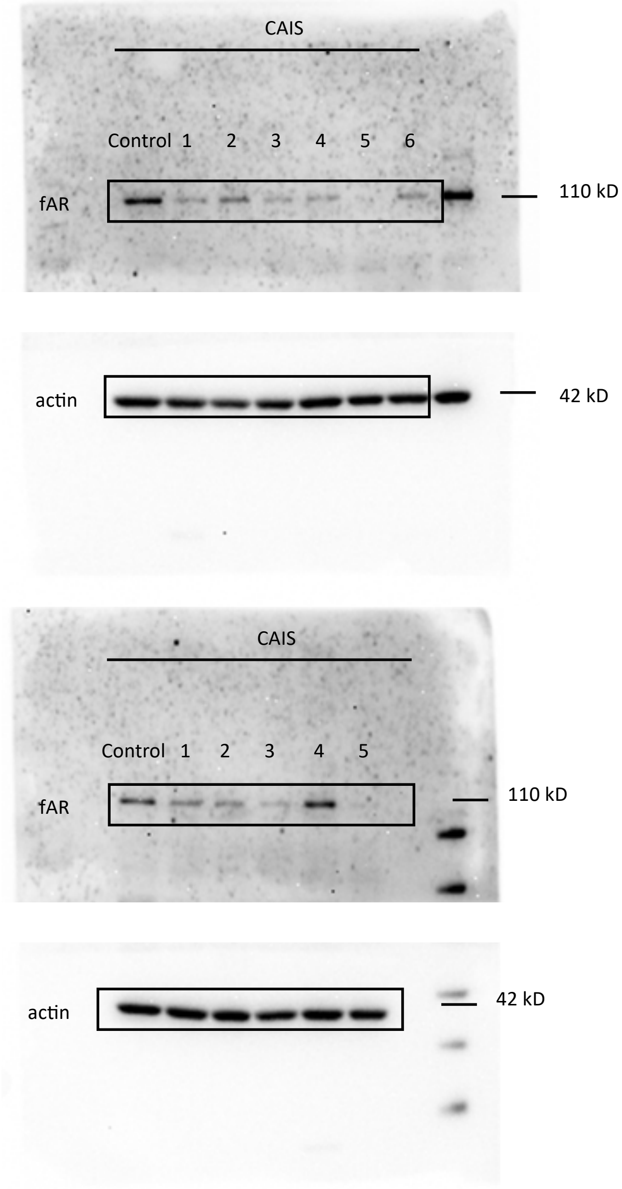

**Figure 1g.**

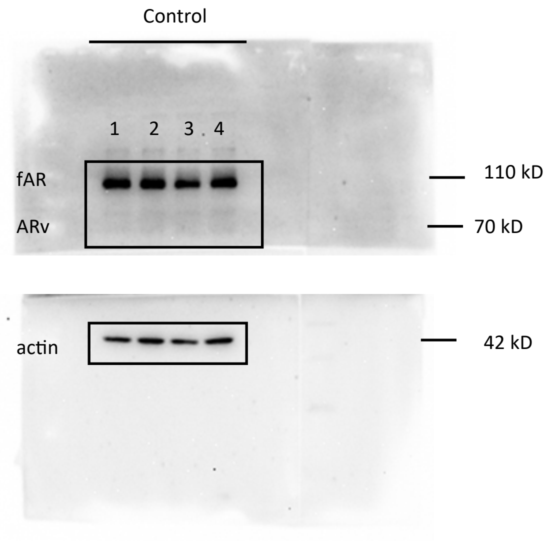


**Figure 1h.**

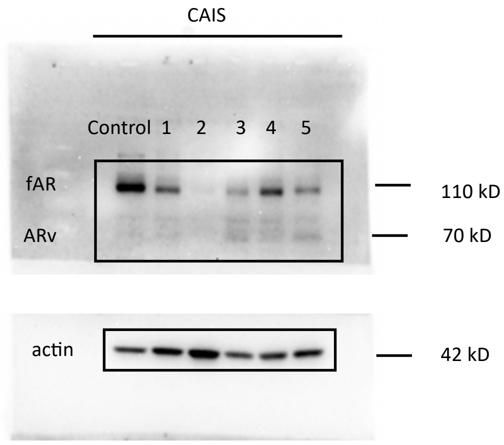


**Figure 1i**

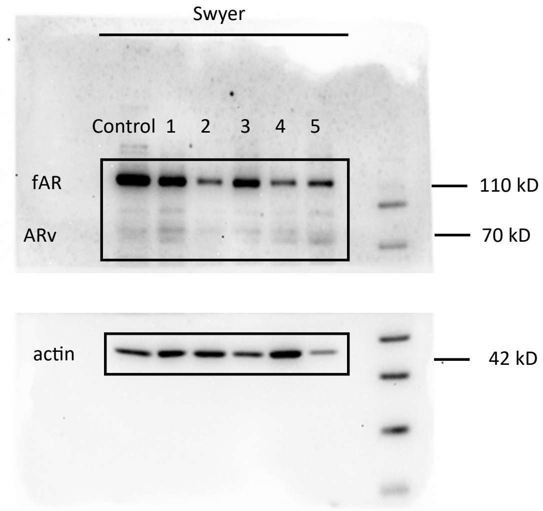

**Figure 1j.**

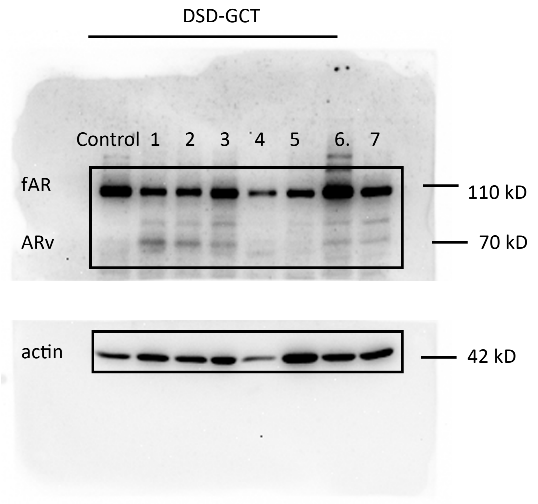

**Figure 3a.**

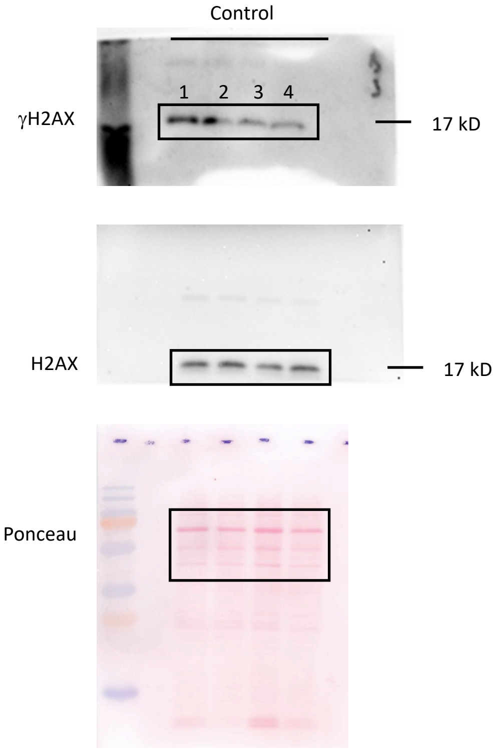


**Figure 3b.**

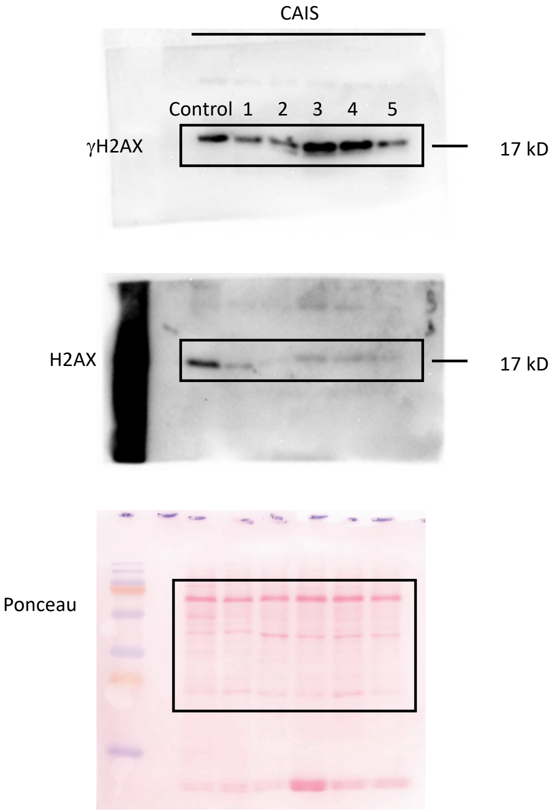

**Figure 3c.**

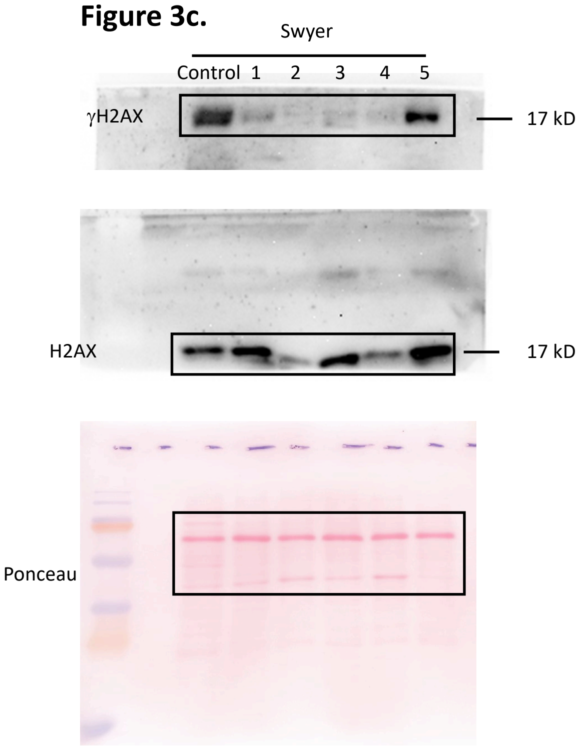


**Figure 3d.**

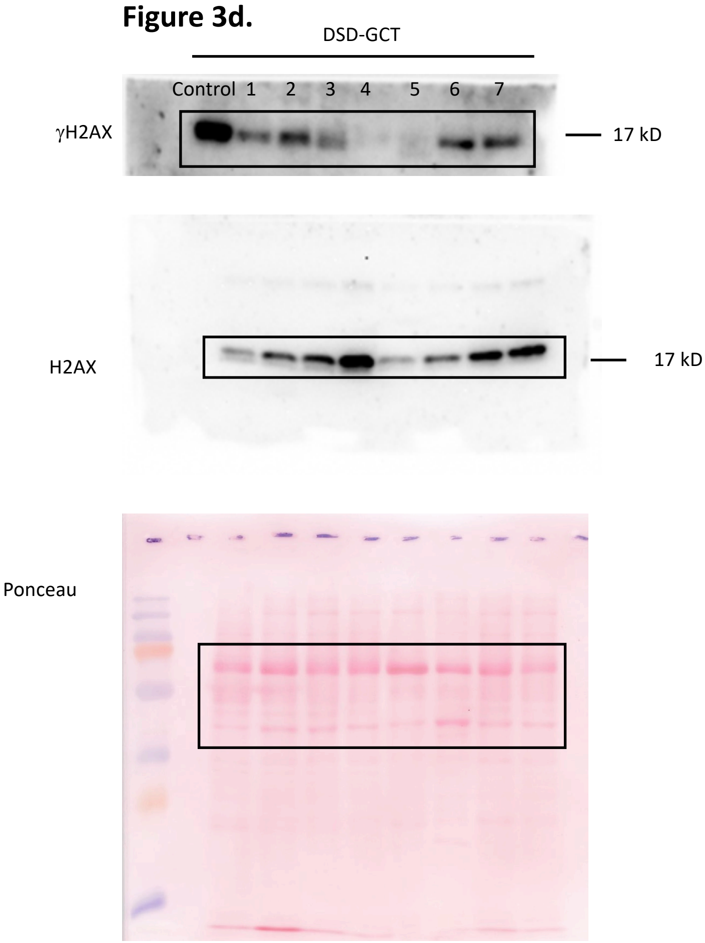

**Figure 4a.**

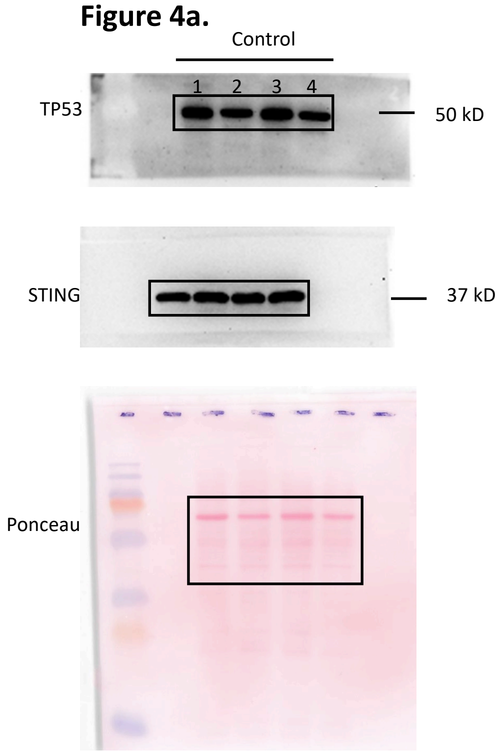


**Figure 4b.**

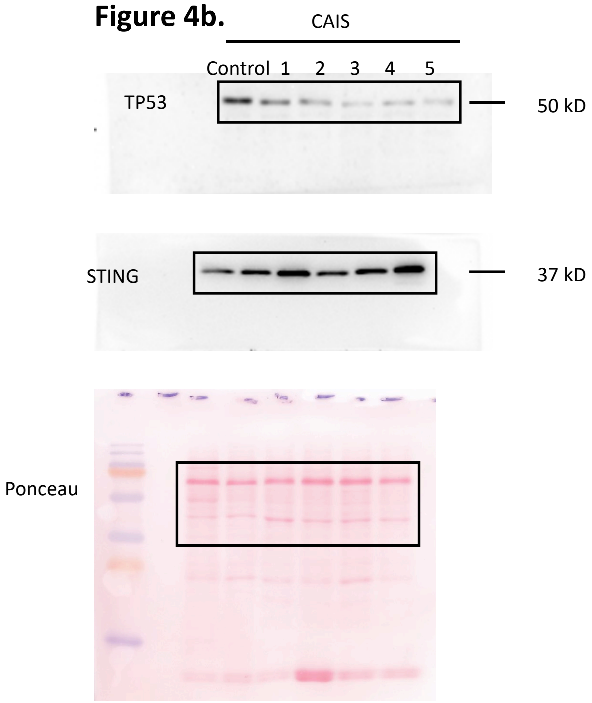

**Figure 4c.**

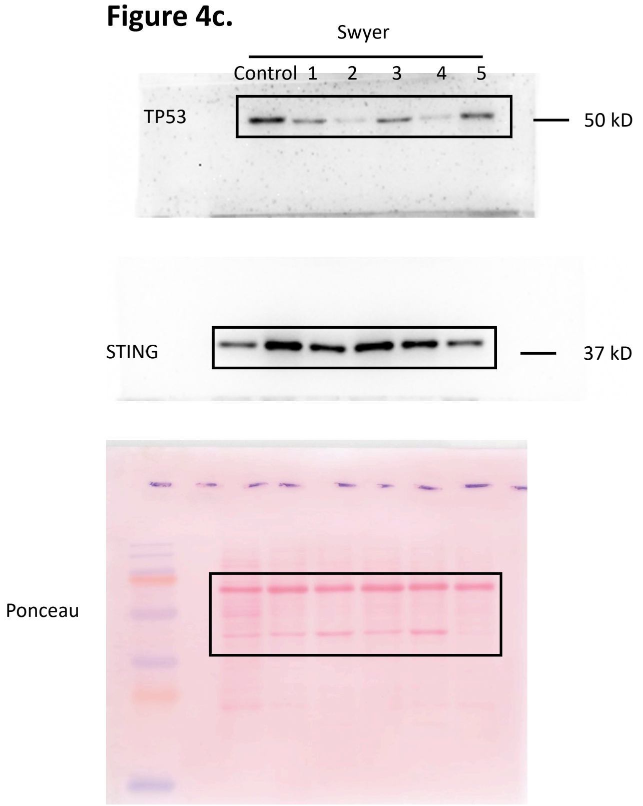


**Figure 4d.**

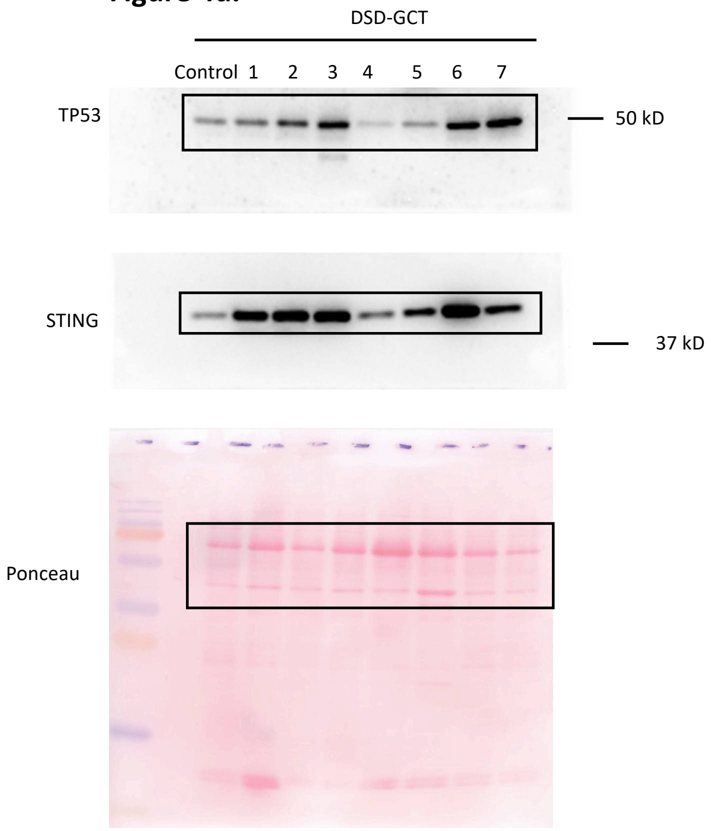

**Figure 4g.**

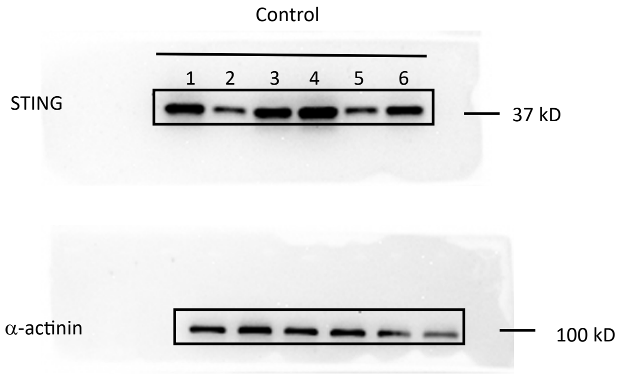


**Figure 4h.**

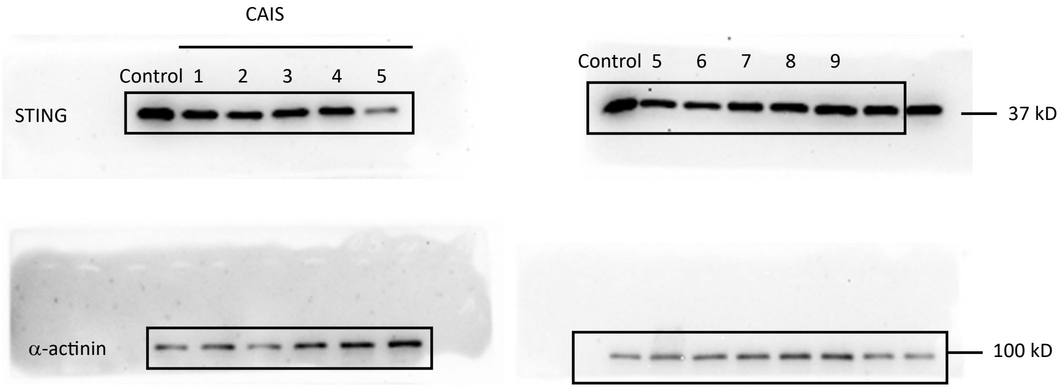

**Figure 5a.**

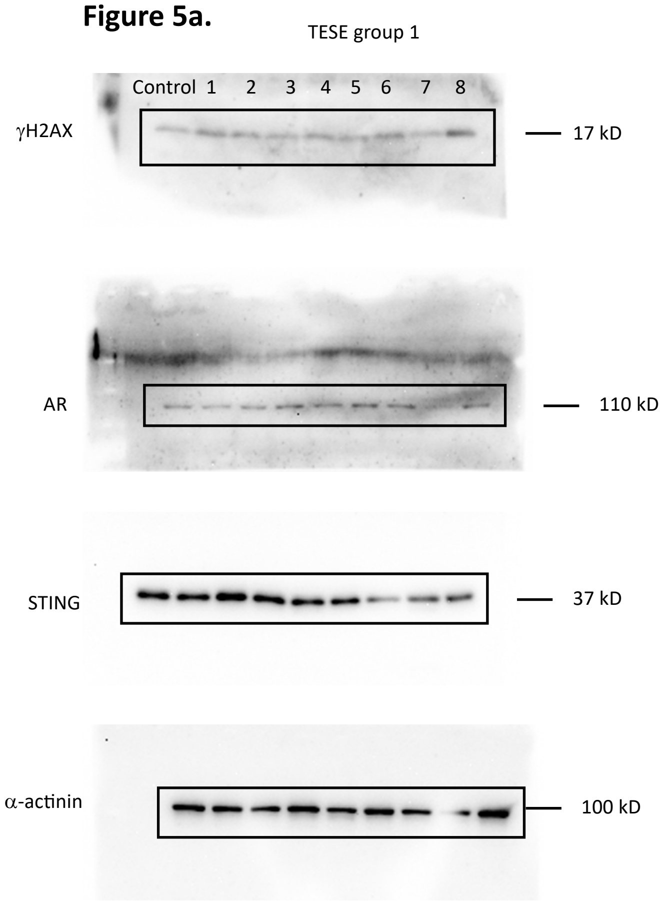


**Figure 5b.**

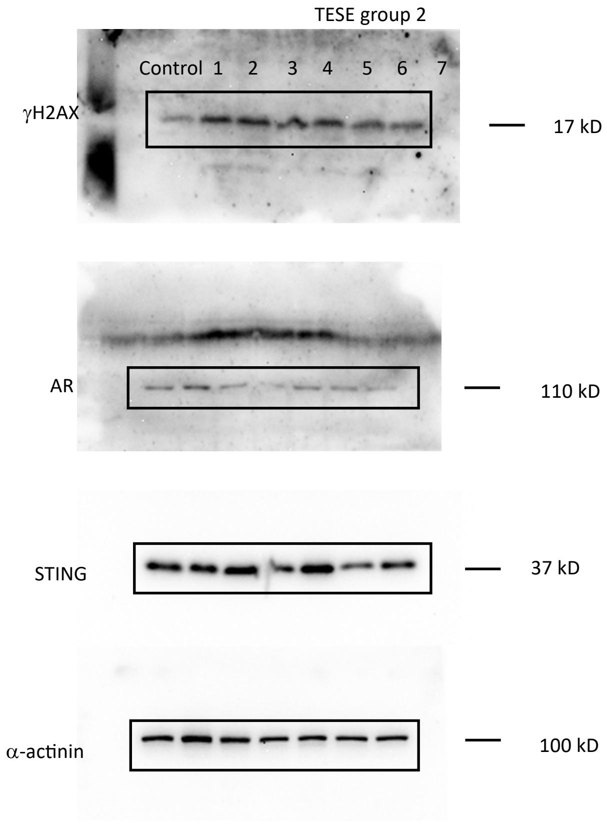

## Figure 5c.

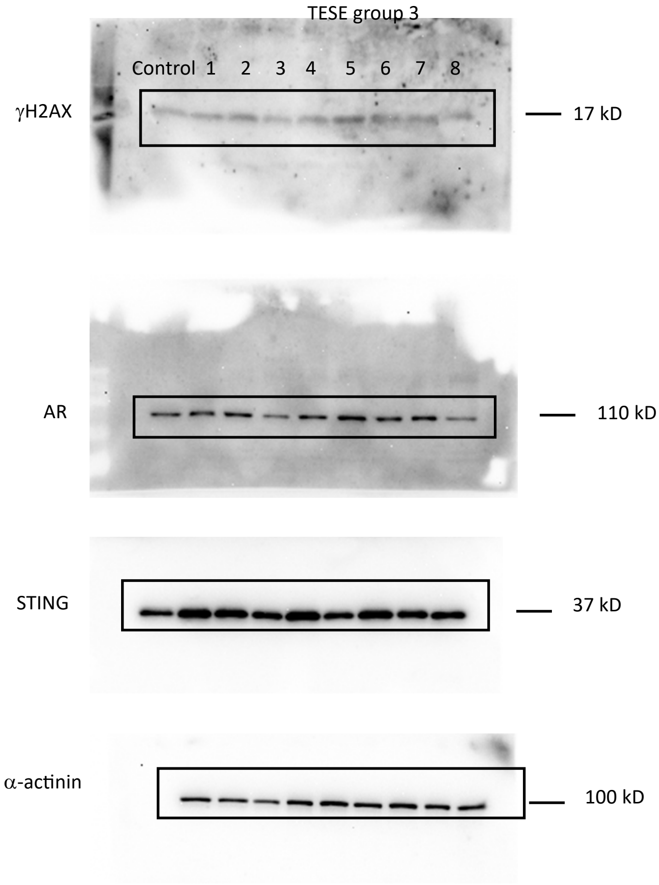


## Figure 5h.

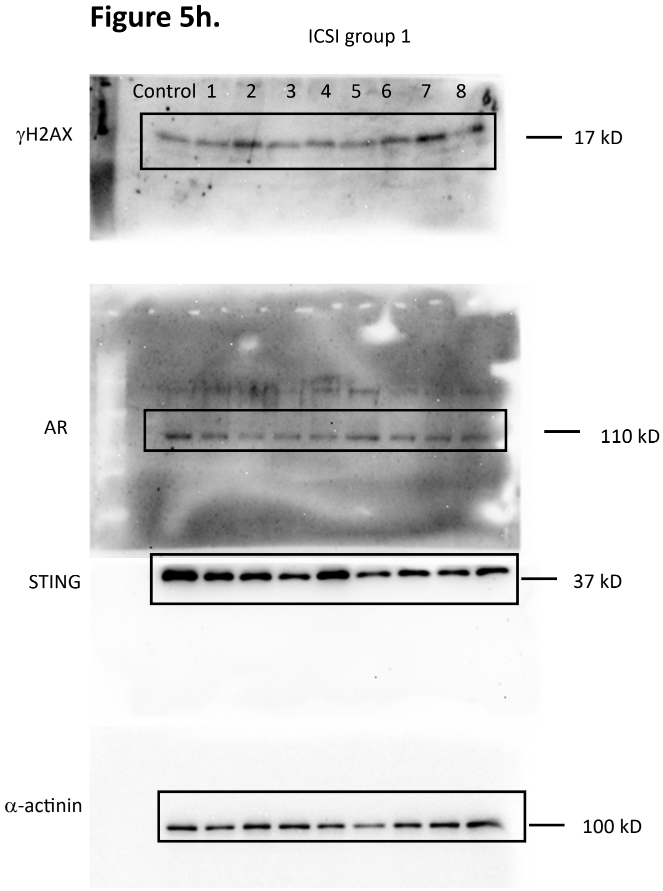

**Figure 5i.**

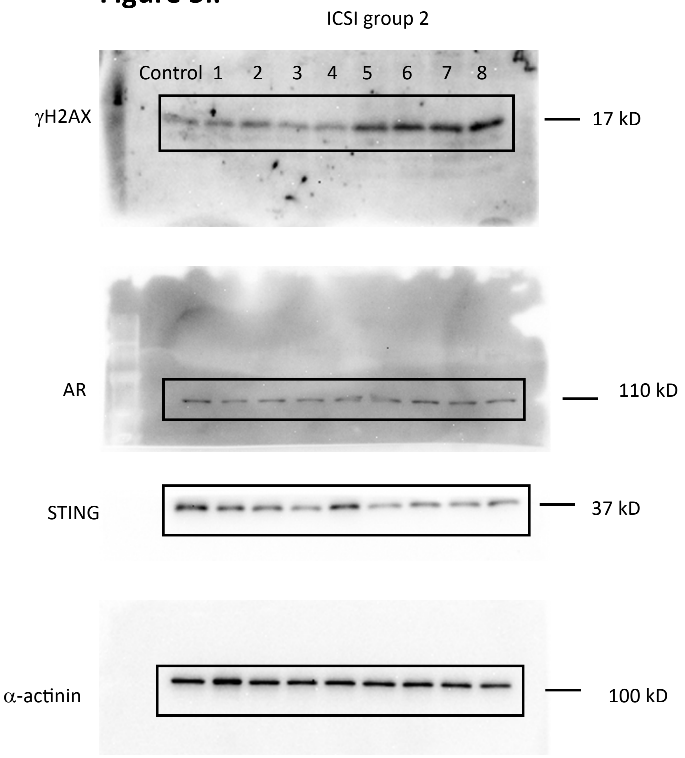
ICSI group 2
Control 1 2 3 4 5 6 7 8
γH2AX
17 kD
AR
110 kD
STING
37 kD
α-actinin
100 kD

**Figure 7a.**

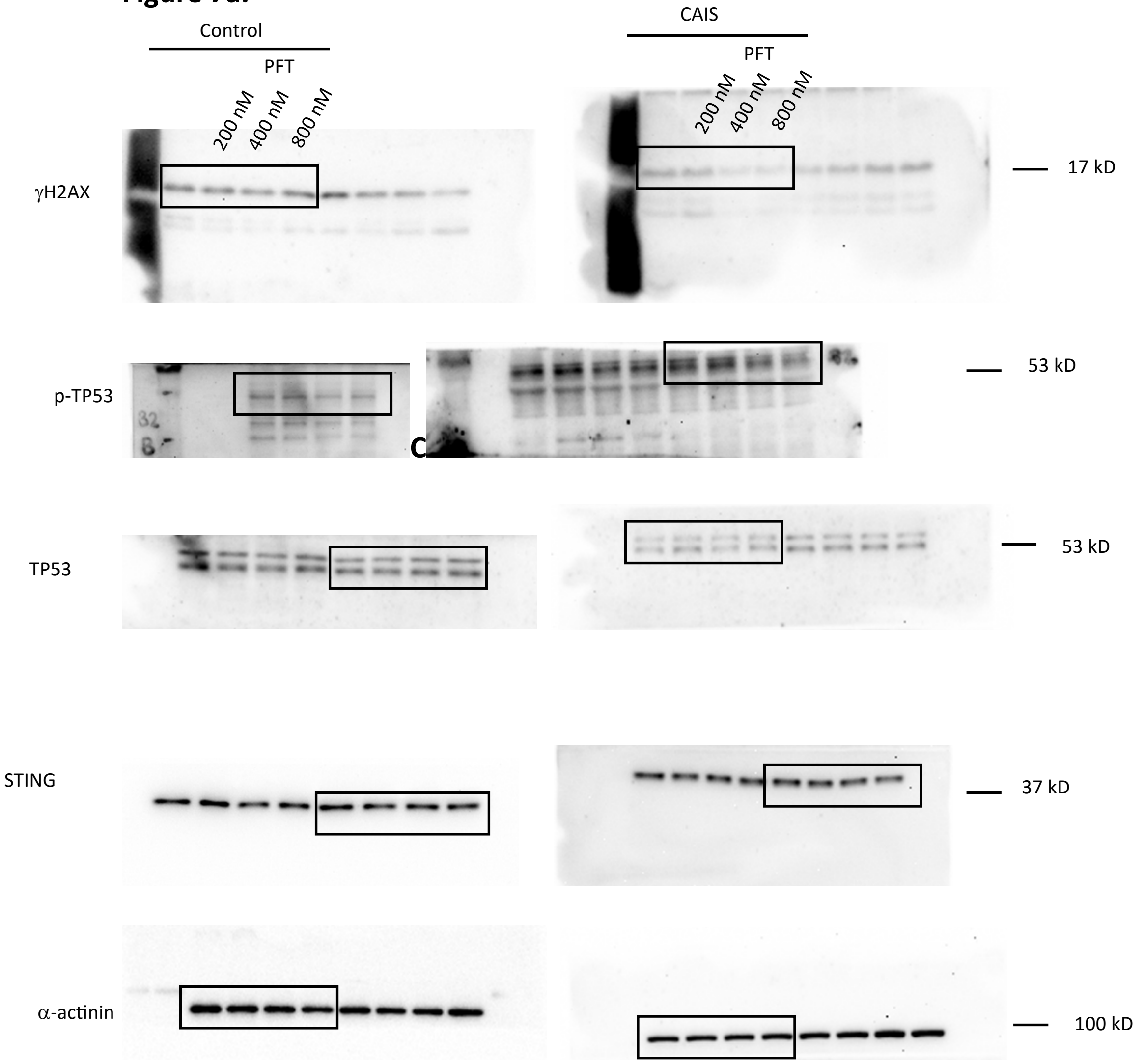
Control
PFT
200 nM
400 nM
800 nM
CAIS
PFT
200 nM
400 nM
800 nM
γH2AX
17 kD
p-TP53
53 kD
TP53
53 kD
STING
37 kD
α-actinin
100 kD

**Figure 8g.**

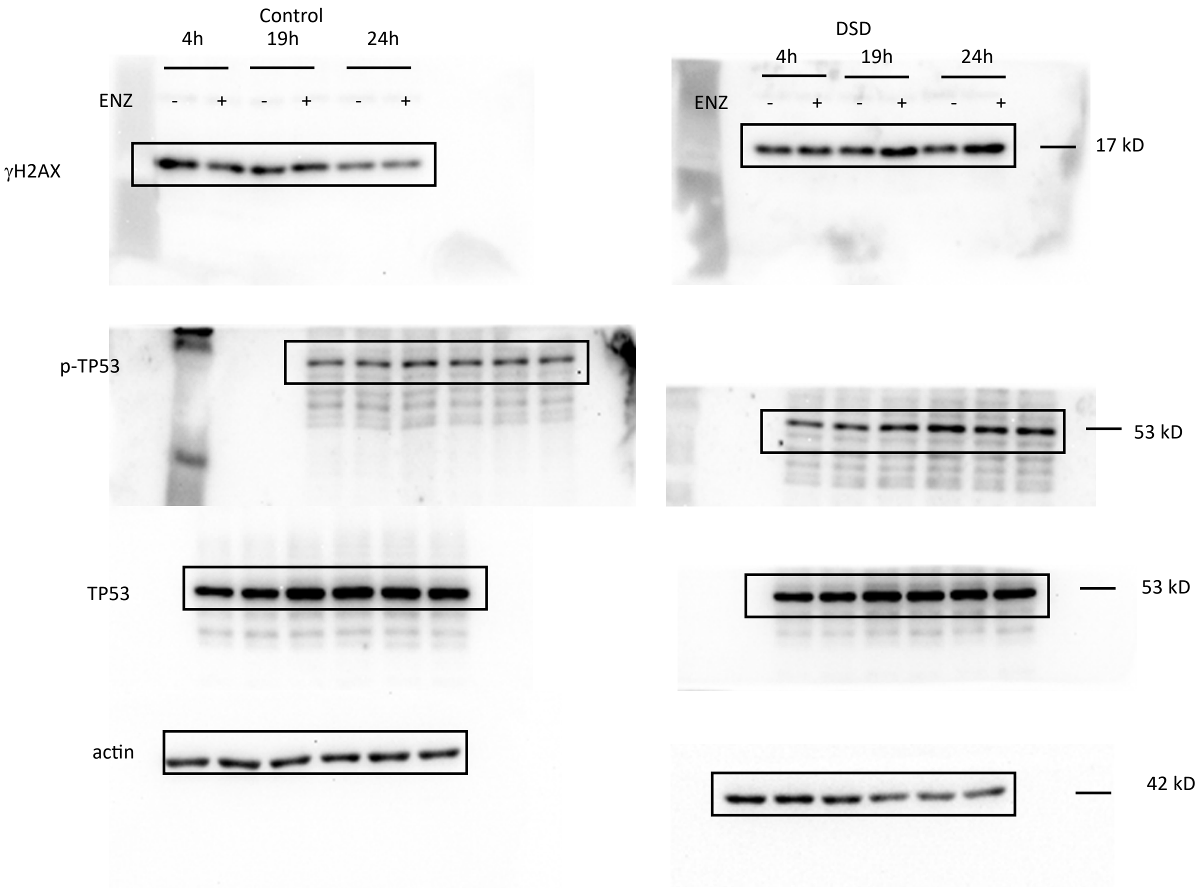


**Figure 8o.**

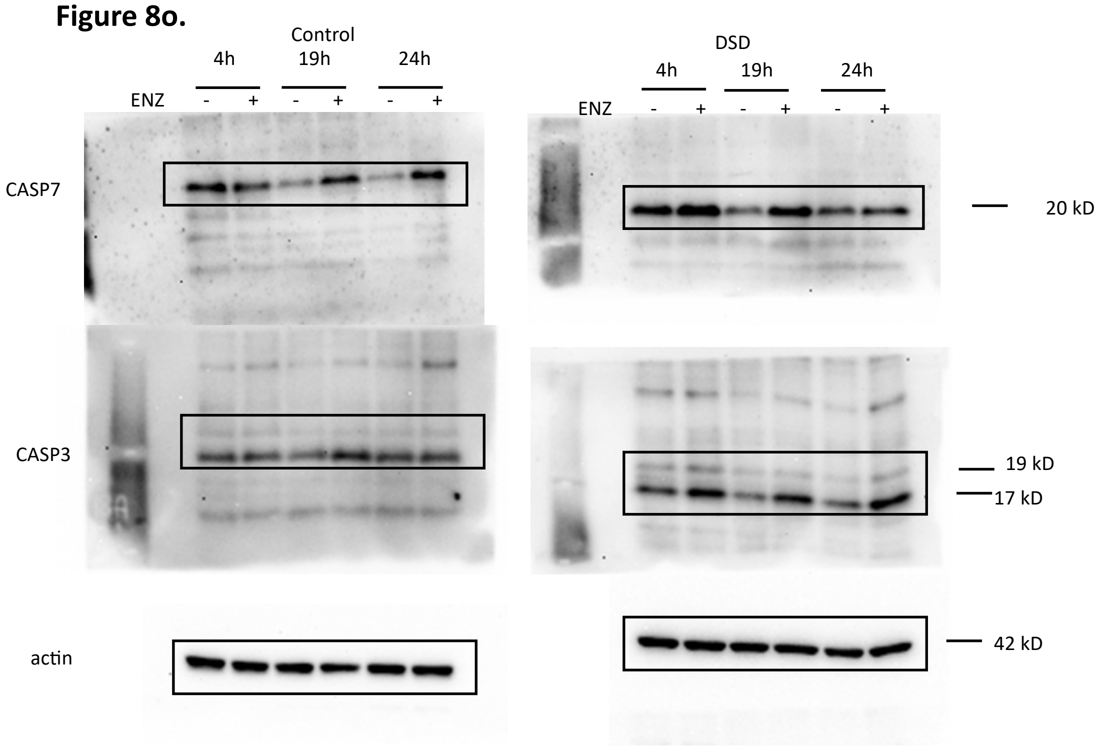

## Extended data figure 6a.

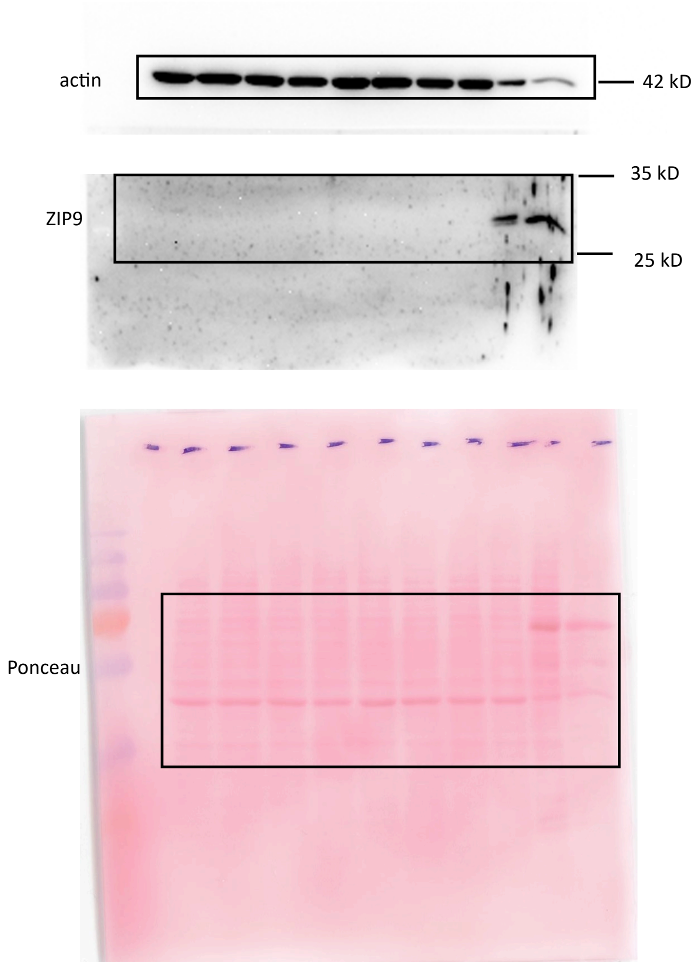


## Extended data figure 6b.

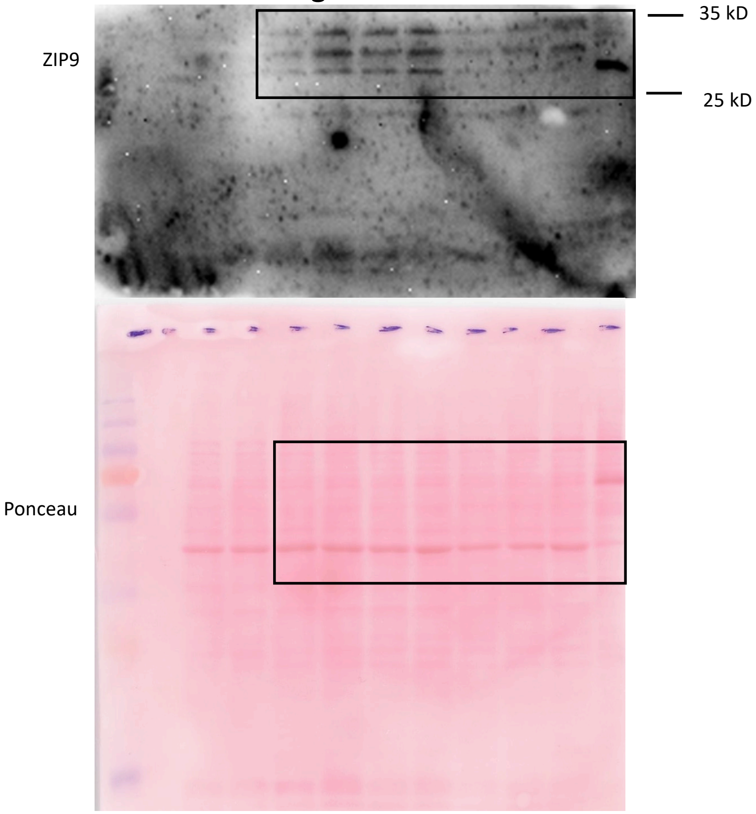

**Quantification of original WB data.**
**Figure 1f**
**fAR-WB**

Control
0.845513307
0.723484126
0.745719284
1.000000002
0.691103577
0.913026387
CAIS
0.241988546
0.490849514
0.252698366
0.224361011
0.113507577
0.286167864
0.657020705
0.694528949
0.381324209
0.989279679
0.28789976

**Figure 1k**
**fAR-WB**

Control
1.318636543
1.056505629
1.149080542
0.999220449
Swyer
0.94
0.59
1.11
0.57
0.6
DSD-GCT
0.830215937
1.507382506
1.172088867
0.563914741
0.969870116
1.146996143
0.859059006
CAIS
0.577677216
0.036154145

0.288525157
0.693666842
0.349544036

**Figure 1l**
**ARv7-WB**

Control
0.999999991
0.99382976
0.986039964
1.01373329

Swyer
1.8161
0.8307
0.6459
1.1815
1.3098

DSD-GCT
8.106238037
9.176987283
5.368373385
1.301941563
2.151044425
3.833459668
2.83189922

CAIS
1.0997264
0.171551538
2.853984576
1.515028693
2.310902029

**Figure 3e**
γH2AX-WB

Control
1.000000002
1.700625863
0.962929953
2.642801586

Swyer
0.263382053
0.115305865
0.105769995
0.267276086

DSD-GCT
0.159729251
0.533538411

0.452001683
0.091712568
0.202482953
0.503974453
0.630484078
CAIS
0.400417117
0.059060084
0.878720343
0.202430442
0.614946605

**Figure 3f**
Total H2AX-WB
Control
0.999999999
1.55412389
0.80301983
1.509022257
Swyer
2.671034805
0.17565499
1.68164279
0.260074372
5.038672181
DSD-GCT
1.570516294
2.065271878
4.093218514
1.000000006
1.078128445
2.815611409
4.114173499
CAIS
0.901543823
0.788255719
3.057987462
1.786409281
0.87338564

**Figure 4e**
STING-WB
Control
1
1.278594292
1.41396148
2.081356313

Swyer
2.812468595
2.526676644
2.950066281
2.311077251
0.930122162
DSD-GCT
4.679380468
4.564257114
5.482656031
1.722937443
2.596822406
4.680517324
2.695661308
CAIS
2.039376203
2.679689056
1.283665091
1.484156982
3.671982692

**Figure 4f**
flTP53-WB
Control
0.999999995
1.085537272
1.22272406
1.003442548
Swyer
0.71692014
0.212310473
0.911799249
0.248079308
1.521646726
DSD-GCT
1.248530681
2.500957385
3.287881796
0.221199571
0.623754136
3.144206769
4.51940337
CAIS
1.476517079
0.829686551
0.423773137
0.369046049

0.350917674

**Figure 4i**

STING-WB

Control

1.200967452
0.846553848
1
0.481717537
1.237277488

CAIS

0.423742603
0.497880607
0.420828126
0.291660489
0.60344288
0.793355995
0.687541163
0.80385431

**Figure 5d**

γH2AX-WB

Control

1.66045866
1.19291582
1.43481417
2.00040009
0.6656339
2.85014866
0.79465597
1.59823942

SGA/SCA

1.34453657
1.77506494
1.57489393
2.21530728
2.03453728
1.41887618

SCO

0.71168572
1.96066165
0.66268754
0.91799233
1.86018352
1.10288867
1.17634509
0.59671673

**Figure 5f**

AR-WB

Control

0.36047517
0.49690652
1.131073
0.69659608
1.28459393
0.58299776
0.4223041
1.19982178

SGA/SCA

1.63721484
0.50628471
0.22200867
0.51999791
0.96367486
0.32339181

SCO

1.69872938
1.36770479
0.56903718
0.9384855
2.12494077
0.88701955
1.86665995
0.54927237

**Figure 5g**

STING-WB

Control

0.56669236
0.50752817
0.69456339
0.45359177
0.57872163
0.21748838
0.45045684
0.65483624

SGA/SCA

0.67874704
0.73845433
0.80052851
0.59067608
0.44579991
1.06940698

SCO
1.97995868
1.22287531
1.00463734
1.2994197
0.95265141
1.52971848
1.58088523
1.32688496

**Figure 5j**
γH2AX-WB
Normal embryogenesis
0.53731396
1.24728237
0.20533577
0.89640717
0.86169506
0.97524489
1.80003299
0.630033
0.90082426
0.92382866
1.55985287
0.33882768
0.42065619
1.49877623
1.36405279
Abnorman embryogenesis
1.37139067
1.45092575
0.72352571
1.03743994
2.40119936
2.27000233
2.47532288
3.95966669

**Figure 5l**
AR-WB
Normal embryogenesis
0.68893966
0.20820502
0.57822585
0.40290261
0.43786212
0.33592318

0.70864215
1.13050305
0.61872937
0.51249466
0.26590751
0.22145574
0.82742558
0.55879113
1.37396373
1.5661175
Abnorman embryogenesis
0.58746718
0.35951712
0.63638821
0.51528845
0.56370784
0.5067873
0.34113578

**Figure 5m**
STING-WB
Normal embryogenesis
0.61124579
0.84018293
1.04722908
0.54284092
0.56792148
0.71096145
0.52717379
0.06711537
0.76401159
0.86999534
0.79646246
0.74692992
0.68673767
0.43059868
1.25142586
0.69122095
Abnorman embryogenesis
0.81943486
0.4338601
0.32952055
0.79684522
0.19593916
0.24261184
0.33750395
0.38066787

**Figure 7b**
p-TP53-WB
Control cell lines
Control
1
1
1
1
1
200 nM
1,05804604
0,77754286
0,9320106
1,29527226
1,61564923
400 nM
0,99893481
0,90219006
1,56615463
1,13629707
0,90416918
800 nM
0,97653974
0,73322244
1,21828289
1,3376543
2,28342229

DSD cell lines
Control
1
1
1
1
1
1
1
200 nM
1,30181326
0,91129892
0,80020744
0,77265682
1,04766173
0,79852646
1,32990101
400 nM

0,97198289
0,74301755
0,82514391
1,03310402
0,84336383
0,77389716
0,78918289
800 nM
0,93186687
0,54383799
0,36436168
0,75324443
1,1132288
0,46699299
0,87199925

**Figure 7c**
TP53-WB
Control cell lines
Control
1
1
1
1
1
200 nM
0,91822313
0,7440024
0,44061458
1,32257088
0,80471059
400 nM
1,26900316
0,79480111
0,39122821
1,46000965
0,7609599
800 nM
1,90717703
0,55130666
0,61141982
1,21460159
0,50499688
DSD cell lines
Control
1
1

1
1
1
1
1
200 nM
1,59383935
0,72264344
1,40898616
0,86569552
2,25290817
1,1026317
0,92861453
400 nM
1,5206207
0,7825922
1,07736735
0,62605312
2,71016449
1,91336614
0,95343652
800 nM
1,33796579
0,45302992
1,31624935
0,58179568
2,30946327
2,48955369
1,17538114

**Figure 7d**
γH2AX-WB
Control cell lines
Control
1
1
1
1
1
1
200 nM

0,821744972
0,509049424
1,283217228
0,968339966
0,775060238
0,42986574

400 nM

0,752514681
1,463411004
1,736992747
0,725176912
0,683327148
0,56898773

800 nM

0,800130981
1,200316421
1,515856978
1,202358705
0,344089546
0,83953649

DSD cell lines

Control

1
1
1
1
1
1
1
1
1

200 nM

0,79664058
0,671219841
0,594692969
0,826365976
0,423198436
0,631832263
1,08753199
1,02313513

1,058402712
400 nM
0,636408255
0,927930376
0,499722195
0,214727937
0,654031639
0,825373718
0,355177925
0,94617232
1,179668047
800 nM
0,405993911
0,722163242
0,193868445
0,2615061
0,966096186
0,507274115
1,02081691
0,959682865

**Figure 7e**
STING-WB
Control cell lines
Control
1
1
1
1
1
200 nM
1,09211329
0,78048209
1,05267362
1,01259696
0,86596477
400 nM
1,04586553
1,18116322
1,11865602
1,15680764
0,99625412
800 nM
0,99977817
0,92284112
1,16334898
0,99363452

0,73071264
DSD cell lines
Control
1
1
1
1
1
1
1
200 nM
1,0452552
0,9605051
0,99623798
0,90537989
0,94219387
1,01798245
0,98804141
400 nM
1,15488664
0,90042567
0,68000649
1,18100505
0,93582343
1,24040105
0,67664025
800 nM
0,96234946
0,85803052
1,04416691
0,89886724
1,1661126
1,45328321
0,49113402

**Figure 8h**
γH2AX-WB
Control cell lines
4h
1,20676678
1,17091072
1,57766706
0,47258311
19h
2,56025185
1,48251133
1,15311946

1,10774559
24h
1,82278177
2,05198982
1,28677443
1,10581644
DSD cell linies
4h
1,08404755
1,11223768
1,00721986
0,72098199
19h
1,47221891
1,53832938
0,89451239
1,83044386
24h
2,56196613
2,63862244
1,25655924

**Figure 8i**
TP53-WB
Control cell lines
4h
1,138705318
1,28845054
1,08396759
1,10033423
19h
0,92130965
0,85154125
0,71171626
0,94655115
24h
1,17074135
1,20839777
1,15904512
1,10024794
DSD cell lines
4h
0,87386407
0,92894076
0,91046121
0,77953259
19h

1,05133797
0,92376371
0,93558522
0,93396081
24h
1,26214512
1,06283385
1,03669576
1,06363615

**Figure 8p**
CASP7-WB
Control cell lines
Control 4h
1
1
1
1
ENZ 4h
1,16926472
0,92807999
1,5575771
2,92411677
Control 19h
0,29668669
0,36598967
0,58258001
0,60485245
ENZ 19h
0,81866401
1,22527953
1,85827753
1,46585164
Control 24h
0,12722666
0,24884007
0,51299821
0,93290778
ENZ 24h
1,35672093
1,48923467
1,3920635
2,4982255

DSD cell lines
Control 4h
1

1
1
1
ENZ 4h
0,74647016
1,55738309
1,15868754
1,08003456
Control 19h
0,46757049
0,33651799
0,16503031
0,17006253
ENZ 19h
0,8519111
1,10241838
1,0516513
0,94938133
Control 24h
0,74063833
0,34622174
0,35119991
0,33308919
ENZ 24h
0,63384491
2,78761036
0,71067118
0,97392521

**Figure 8q**

CASP3 17kD-WB
Control cell line
Control 4h
1
1
1
1
ENZ 4h
1,15581446
0,82120907
1,52799713
0,92145613
Control 19h
0,46739323
0,37841765
1,12161188
0,5464994

ENZ 19h
1,02446757
0,9609261
1,04721947
1,11510527
Control 24h
0,2750544
0,37799086
0,59864945
0,78220866
ENZ 24h
1,24068762
0,86469116
1,19715971
1,08473167

DSD cell lines
Control 4h
1
1
1
1
ENZ 4h
1,743992
1,63197368
1,11921152
1,57485679
Control 19h
1,29123137
0,53463448
0,3244214
0,85306009
ENZ 24h
1,30053138
2,16048326
1,10334565
1,11997061
Control 24h
0,77460049
0,32336535
0,54180688
0,4844825
ENZ 24h
1,32090182
1,27485524
1,17617174
1,37966925

**Figure 8r**
CASP3 19kD-WB

Control cell lines
Control 4h
1
1
1
1
ENZ 4h
1,49271765
0,89031433
2,29444933
0,62952808
Control 19h
0,5598253
0,35427151
1,01067283
0,28375425
ENZ 19h
1,17841131
0,92148117
0,76837614
0,55920031
Control 24h
0,31004384
0,50083512
0,67639824
0,59035858
ENZ 24h
1,59952117
0,790322
1,49473987
0,67243218

DSD cell lines
Control 4h
1
1
1
1
ENZ 4h
1,93541544
1,53428234
1,0451544
2,22434646

Control 19h
1,03261924
0,38475448
0,32386807
1,05602506
ENZ 19h
1,89063637
0,9212424
0,51238917
1,29671529
Control 24h
0,45714559
0,16564104
0,32132042
0,62058074
ENZ 24h
2,44189606
1,65211175
0,84881162
1,44563172

**Original qPCR data.**
**Figure 2c**
*IFN*$\alpha$-qPCR
Control
0.518486
0.358665
1.021215
CAIS
5.685978
4.7796885
4.406565
0.874961
1.3520805
0.6222025
1.659996

**Figure 2d**
IL1$\beta$-qPCR
Control
1.082632
0.454215
0.633577
0.722807
CAIS
1.300326
1.29397

1.545619
1.314072
0.776499
1.590943
1.125681

**Figure 2e**
*MX2*-qPCR

Control
0.945517
0.678174
0.834382
0.614946
CAIS
1.282754
0.838699
1.421701
1.303399
1.274353
0.842939
0.943692

**Figure 2f**
*IFN$\beta$*-qPCR

Control
1
0,4814
1,4483
1,3722
Swyer
1,7631
0,8478
1,2469
1,4842
7,8064
DSD-GCT
1,4793
3,153
122,7684
2,6668
79,4348
1,6468
2,3949
CAIS
0,9543

0,1286
0,6549
0,7967
0,7939

**Figure 2g**
*IL6*-qPCR

Control
1
0,5474
0,444
0,9358
Swyer
6,1817
0,6459
4,9468
1,2438
0,1451
DSD-GCT
16,7179
33,4533
19,3126
64,3541
66,0059
22,6233
52,3235
CAIS
3,8088
3,116
62,5875
49,0305
3,4506

**Figure 2h**
*ISG56*-qPCR

Control
1
1,6398
1,3726
4,0117
Swyer
4,9353
15,2315
7,601
16,5383

0,7444
DSD-GCT
2,3176
2,9289
0,8166
8,8622
9,956
3,7261
5,3209
CAIS
1,7815
9,6799
14,3101
3,0726
1,4829

**Figure 2i**
*ISG15*-qPCR
Control
1
1,268
0,8303
1,2135
Swyer
15,3234
74,0209
12,1463
71,7785
1,11
DSD-GCT
12,7597
68,1663
32,8733
33,5579
76,4816
15,8843
71,401
CAIS
6,6102
58,1488
32,0691
4,3792
6,576

**Figure 2j**
*IL1$\beta$*-qPCR
Control

1
0,7516
0,6315
0,846
Swyer
0,1526
0,0851
0,1294
0,0581
0,0733
DSD-GCT
0,862
3,5721
0,7947
0,5136
3,7712
0,9661
3,3054
CAIS
0,1437
0,0479
1,0316
1,3395
0,1132

**Figure 2k**

*IFN*$\alpha$-qPCR
Control
1
0,376
0,5559
0,4579
Swyer
0,065
0,1425
0,1264
4,2411
DSD-GCT
0,4586
0,0477
35,788
0,5654
12,5835
0,5822
0,0684
CAIS
2,3121

0,7939
1,2461
1,3387

**Figure 2l**
*MX2*-qPCR

Control
1
1,3837
1,3105
3,503
Swyer
0,1801
1,5044
0,1628
1,5584
0,2854
DSD-GCT
0,451
2,5538
1,0396
1,1126
2,7845
0,4538
1,8056
CAIS
0,5107
0,9827
1,0928
0,3119
0,4159

**Figure 3g**
*H2AX*-qPCR
Control
1
2,9402
5,0022
2,0296
Swyer
3,2288
2,7663
2,8025
3,8369
0,9653
DSD-GCT

2,6683
3,027
2,6808
1,4054
5,336
2,3437
2,8284

CAIS

1,5002
1,8305
1,9388
2,5096
2,0453

**Figure 5e**

*H2AX*-qPCR

Control

1.1
1.24
1.12
1.32
2.15
1.4
1.18
1.132858872
0.862136

SGA/SCA

1.47
1.6
1.23
1.23
1.039986
1.156743

SCO

1.36
1.74
1.38
2
2.21
2.77
3.74
1.460424

**Figure 5k**

*H2AX*-qPCR

Normal embryogenesis

1.18
1.23
1.156743
1.1083
0.7646
1.1955
0.892862
0.9497
0.946
0.8886
0.975
1.0155
0.8728
1.1729
1.245
1.0114
1.24
1.12
1.4
1.18
1.132858872
0.862136

Abnormal embryogenesis

2.15
4.92
1.039986
0.6557
1.267
1.7378
0.703
1.03
1.15
1.1
1.460424

**Figure 5n**

*IFNβ*-qPCR

Control

0.4756
0.448
0.2834
0.326
1.3282
0.4631
0.4752
0.4225
1.4068

SGA/SCA
1.0586
1.2569
0.1737
0.2145
1.7659
1.2123
SCO
0.32
0.1476
0.3438
1.5201
2.1244
2.1722
5.6668
2.3207

**Figure 5o**

*IFN$\beta$*-qPCR

Normal embryogenesis
0.4752
0.1737
1.2123
1.5582
0.59735
1.6823
0.6981
1.5604
0.15755
0.16155
0.2491
0.36275
0.11745
0.34535
0.5142
2.0574
1.7251
0.448
0.2834
0.4631
0.4752
0.4225
1.4068
Abnormal embryogenesis
1.3282
9.6247
1.7659

1.65735
3.693
4.2995
0.1665
0.21355
2.68285
0.4756
2.3207

**Figure 5p**
*IFN*$\alpha$-qPCR
Control
0.3522
0.3032
0.1886
0.2907
0.3076
0.3691
0.2537
0.9094
SGA/SCA
0.5384
0.6948
0.0773
0.0957
0.6761
0.6865
SCO
2.5672
0.1208
2.8493
1.6379
2.4007
3.0694
9.4266
1.9003

**Figure 5q**
*IFN*$\alpha$-qPCR
Normal embryogenesis
0.3691
0.0773
0.6865
0.8391
0.4263
1.1652
0.4899

1.2914
0.1907
0.2241
0.5059
0.7522
0.1937
0.7438
1.5116
1.3576
0.3032
0.1886
0.3691
0.2537
0.9094

Abnormal embryogenesis

1.339
0.3004
0.6761
1.6654
5.3736
3.6813
0.3741
0.3906
2.844
0.3522
1.9003

**Figure 5r**

*IL1$\beta$*-qPCR

Control

1.1126
0.3498
0.1759
0.7622
1.1321
0.5265
0.3682
0.7216

SGA/SCA

26.199
3.2591
1.9454
2.2633
4.5491
2.9527

SCO

1.1244

1.2292
1.0398
0.9095
0.9335
0.707
0.7516
2.2802

**Figure 5s**

*IL1*β-qPCR

Normal embryogenesis

0.5265
1.9454
2.9527
0.449
0.2255
0.543
0.4833
0.3231
0.4453
0.8621
0.1215
0.6176
0.7049
0.6939
0.9086
0.9255
0.3498
0.1759
0.5265
0.3682
0.7216

Abnormal embryogenesis

0.4336
0.5137
4.5491
0.3547
1.4476
1.7585
0.7733
1.4498
4.3702
1.1126
2.2802

**Figure 7f**

*IL6*-qPCR

Control cell lines
Control
1
1
1
1
1
200 nM
0,45566877
0,73713499
0,88058414
2,41499257
0,73484722
400 nM
0,28203611
0,75123452
0,8881218
0,41467426
1,32655602
800 nM
0,61243809
0,91360005
1,31263191
0,42784265
1,71030715

DSD cell lines
Control
1
1
1
1
1
1
1
200 nM
0,99675134
0,40028798
0,61777867
0,51997303
0,24500418
0,95629195
400 nM
0,67865516
0,4730449
1,05053836
0,72015829

1,07695674
0,24726434
0,86879364
800 nM
0,60380009
0,6406505
1,36982709
1,23940297
1,04404842
0,48110531
1,03190877

**Figure 7g**
*IL12α*-qPCR

Control cell lines
Control
1
1
1
1
1
200 nM
1,04888249
0,870342
0,96175425
0,94787032
0,96682586
400 nM
1,07855853
0,77738351
0,91888997
0,9031289
1,07785317
800 nM
0,95775078
0,92880392
0,99548652
0,98911467
1,22206547

DSD cell lines
Control
1
1
1

1
1
1
1
200 nM
0,98303224
0,86671726
1,0591446
0,97501106
0,93827742
1,28683159
400 nM
1,03649579
0,91407429
1,06908207
0,99890394
0,88426973
0,83631068
1,77514023
800 nM
0,97944918
0,7699927
0,85398272
0,85141354
0,75667036
0,71832466
1,91165824

**Figure 7h**
*IFNβ*-qPCR
Control cell lines
Control
1
1
1
1
200 nM
1,21146505
0,78854137
2,78702805
1,08172637
400 nM
0,96265808
0,64799757
1,368473
0,93243919
800 nM

1,2321145
1,14559825
1,11905655
0,96047364

DSD cell lines

Control

1
1
1
1

200 nM

0,88521806
1,40280638
0,65431506
0,96921096

400 nM

0,46158107
0,50697465
0,76494695
0,32547509

800 nM

0,42808849
0,75100861
0,74959425
0,65427455

**Figure 8k**

*IFN*$\alpha$-qPCR

Control cell lines

4h

0,70901074
0,8488231
1,88090816
1,04452163

19h

3,11996817
0,66970987

24h

1,87917366
0,51560127
2,55784016
0,93479281

DSD cell lines

4h

0,80947683
0,6483492

0,65576838
19h
1,64669171
0,98377744
0,50692729
1,96335833
24h
2,2297844
2,13198895
1,81696059
2,62431242

**Figure 8l**
*IFNβ*-qPCR
Control cell lines
4h
1,13925935
1,01819935
1,88532474
0,91639504
19h
2,01340959
1,0990104
1,84410655
1,03413733
24h
3,69961432
1,59859965
3,15049729
1,5239757
DSD cell lines
4h
1,19246712
1,08371177
0,70399159
1,09954232
19h
1,43769434
1,35198749
0,80341703
2,18509312
24h
1,82836322
1,73908264
2,00559666
3,1805163

**Figure 8m**
*IL1β*-qPCR
Control cell lines
4h
0,62598017
0,79283216
0,68357478
0,86565291
19h
1,20853106
1,71838225
1,02747561
0,85364916
24h
1,28277645
1,7190931
1,01385212
DSD cell lines
4h
0,55961101
0,79698945
0,57100742
0,67800401
19h
0,96688146
1,03227127
0,75245017
0,85024912
24h
1,2771299
1,29339054
0,94097622
0,85626829

**Figure 8n**
*MX2*-qPCR
Control cell lines
4h
0,84138312
1,00697013
1,14000264
0,9764055
19h

1,37286435
1,07871944
0,64913269

1,28071615

24h

0,90585953

0,98349871

0,75786216

4,54412862

DSD cell lines

4h

0,9883245

0,90708751

0,68469143

0,75616592

19h

1,12215441

1,11093671

0,65267036

1,17191493

24h

1,30068863

1,40807749

0,91898716

1,00759116

**Extended data figure 1a**

*AR*-qPCR

Control

1.3803

1.14

1.9822

1.6293

0.4267

0.7514

1

0.6089

0.4393

0.6862

1.3997

Swyer

1.2885

1.1277

2.2399

1.6202

0.8079

1.523

2.6681

DSD-GCT

1.0356
0.3596
0.8833
0.5258
1.5239
CAIS
0.2622
2.1123
2.399
0.9303
1.0236
2.7776
0.0943
0.1669
1.1976

**Extended data figure 1b**
*AR*-qPCR
Control
1
1,2302
1,1919
1,0787
Swyer
21,6367
11,8081
20,0164
13,3769
2,6768
DSD-GCT
3,1476
2,8817
0,337
4,4613
8,536
4,1826
7,1682
CAIS
1,3568
5,89
38,657
10,3711
1,5342

**Extended data figure 1c**
*IFNγ*-qPCR
Control

2.061763
0.597566
0.64233
0.335078
CAIS
0.606182
0.275628
0.135173
0.254271
0.422398
0.605095
0.163517

**Extended data figure 1d**
*IL12α*-qPCR
Control
1.662264
1.01809
1.197894
1.661986
CAIS
1.144047
1.152959
1.180253
1.013559
1.113882
1.070341
1.136228

**Extended data figure 1e**
*TNF*-qPCR
Control
1.259829
1.774475
1.154483
1.661148
CAIS
1.347851
0.275722
2.149393
2.293354
2.054305
4.238574
1.80758

**Extended data figure 1f**
*MX1*-qPCR

Control
1.737338
1.333777
2.6737
1.322749
CAIS
3.582031
1.505247
1.244791
0.889015
4.133505
0.287546
1.53784

**Extended data figure 1g**
*IFNGR1*-qPCR
Control
0.847987
0.715674
0.848395
0.821328
CAIS
0.489841
0.868234
0.539503
0.989286
0.531892
0.265495
0.272953

**Extended data figure 1h**
*IFI16*-qPCR
Control
0.584485
0.692216
0.719891
0.61496
CAIS
0.710303
0.385675
0.716976
0.929179
0.915297
1.09804
0.553942

**Extended data figure 1i**

*IFITM2*-qPCR

Control

1.583146

1.1433787

1.50558

0.978456

CAIS

0.921514

1.689018

1.035985

1.107298

0.774982

0.657744

0.625365

**Extended data figure 4a**

*MX2*-qPCR

Control

1.4012

0.714

0.4581

0.8229

1.0918

0.8485

0.3263

1.1603

SGA/SCA

3.3386

1.8252

1.842

4.882

1.7572

SCO

0.8762

0.4517

0.8719

1.0091

0.7371

0.8747

0.5373

1.9181

**Extended data figure 4b**

*ISG15*-qPCR

Control

0.4249

0.5038

0.1632
0.4802
1.0977
0.1613
0.2388
0.3526
SGA/SCA
1.2179
1.428
1.2792
1.7733
0.926
SCO
0.4451
0.2985
0.3847
0.4324
0.5405
0.4139
0.3861
0.3697

**Extended data figure 4c**
*ISG56*-qPCR
Control
0.361
0.3202
0.0859
0.2997
0.9793
0.1136
0.0827
0.3656
SGA/SCA
0.9415
0.9923
0.7378
1.8834
1.1584
SCO
0.2877
0.19
0.2018
0.2016
0.269
0.1951
0.2119

0.3566

**Extended data figure 4d**
*MX2*-qPCR
Normal
0.8485
1.7572
0.6106
0.481
1.0663
0.4462
0.5719
0.6226
0.767
0.6839
0.6708
0.64
0.7098
1.016
1.0052
0.714
0.4581
0.8485
0.3263
1.1603
Abnormal
0.4624
0.4126
4.882
0.3832
1.6249
2.6746
0.9582
2.5837
1.4012
1.9181

**Extended data figure 4e**
*ISG15*-qPCR
Normal embryogenesis
0.1613
1.428
0.926
0.2448
0.2301
0.8308
0.1575

0.1565
0.5764
0.6574
0.15
0.3657
0.2154
0.2487
0.3133
0.3057
0.5038
0.1632
0.1613
0.2388
0.3526
Abnormal embryogenesis
0.1146
0.1674
1.7733
0.1972
0.6037
0.2721
1.9798
0.4249
0.3697

**Extended data figure 4f**
*ISG56*-qPCR
Normal embryogenesis
0.1613
1.428
0.926
0.2448
0.2301
0.8308
0.1575
0.1565
0.5764
0.6574
0.15
0.3657
0.2154
0.2487
0.3133
0.3057
0.5038
0.1632

0.1613
0.2388
0.3526
Abnormal embryogenesis
0.1146
0.1674
1.7733
0.1674
0.1972
0.6037
0.2721
1.9798
0.4249
0.3697

**Extended data figure 5a**
*IFN*$\alpha$-qPCR
Control cell lines
Control
1
1
1
1
200 nM
2,261857
0,69358447
2,81169072
1,54654586
400 nM
1,82727297
0,49432953
1,21167088
0,67108994
800 nM
1,91333798
0,77959805
0,95642239
0,36771227
DSD cell lines
Control
1
1
1
1
1
200 nM
0,64078681

1,45836184
0,72825507
1,02337646
400 nM
0,26118755
0,38229334
1,85935603
1,45291114
0,22380471
800 nM
0,25634295
0,94621114
1,72697775
0,99631931
0,66660452

**Extended data figure 5b**
*IFNγ*-qPCR
Control cell lines
Control
1
1
1
1
1
200 nM
0,56759864
0,61600309
0,7802363
0,81762242
1,05272234
400 nM
0,49499375
0,42521506
0,80736009
0,71782989
1,46088818
800 nM
0,70276735
0,57116535
1,35847318
0,70712299
1,94216712
DSD cell lines
Control
1
1

1
1
1
1
1
200 nM
1,32205464
0,94472521
0,9810939
0,65382044
0,54101218
0,78034479
400 nM
0,92470571
0,86159593
1,11902992
0,74626523
1,0834933
0,52924328
0,39863603
800 nM
0,81265276
1,05563908
1,1801122
1,11768198
1,30386273
0,92030858
0,58703034

**Extended data figure 5c**

*IL1β*-qPCR
Control cell lines
Control
1
1
1
1
1
200 nM
0,77421609
1,2129531
1,50479192
2,72825591
1,06539756
400 nM
0,57487417
1,04832426

1,91398271
0,91464553
1,21225279
800 nM
0,95536104
0,97454669
2,17056018
0,8647424
1,28866887
DSD cell lines
Control
1
1
1
1
1
1
1
200 nM
1,57046959
1,04485334
1,28471749
1,03709137
0,72970522
1,68773622
400 nM
1,47162418
1,09404859
1,76031763
1,17958055
1,4376625
0,65460925
6,87918516
800 nM
1,39132366
1,47183108
1,78240491
1,60896159
1,25362477
0,95817738
7,60025907

**Extended data figure 5d**

*TNF*-qPCR

Control cell lines
Control
1

1
1
1
1
200 nM
0,79889115
0,68090098
0,91908355
0,76471742
400 nM
0,53618322
0,59183453
1,0263042
0,64222019
1,09980789
800 nM
0,86347641
0,52659098
0,72424148
1,05332376
DSD cell lines
Control
1
1
1
1
1
1
1
200 nM
1,22501896
0,76574361
0,8970577
1,05239393
0,8382264
1,23479921
400 nM
1,02002522
0,85809939
1,07529429
1,2882308
0,92292234
1,16470455
235,786736
800 nM
0,93466656
1,06413658

1,11261798
0,9706694
2,63302352
0,83111735
170,718153

**Extended data figure 5e**
*MX1*-qPCR
Control cell lines
Control
1
1
1
1
1
200 nM
0,98924392
0,92037895
0,90842404
0,76299222
1,07032305
400 nM
3,48871693
0,97813595
0,83354924
1,07888631
0,97681523
800 nM
0,96978347
0,88912294
0,78094408
1,10031837
0,86232001
DSD cell lines
Control
1
1
1
1
1
1
1
200 nM
1,03638697
1,059825
0,86969107

0,94450317
1,21561078
1,72434856
400 nM
0,94982527
1,00205043
0,82603653
0,96366575
0,80759388
1,32707905
40,5448709
800 nM
0,89325996
0,98127194
0,8852517
0,85117175
1,18213243
0,9465354
32,0409576

**Extended data figure 5f**
*MX2*-qPCR
Control cell lines
Control
1
1
1
1
200 nM
1,04776033
0,98067968
0,86167707
0,78403674
1,17183617
400 nM
1,5511542
1,05931253
0,98226684
0,96743778
0,9820026
800 nM
0,90001852
0,71031104
0,62692434
0,94055666
1,02147549
DSD cell lines

| | |
|---|---|
| Control | |
| | 1 |
| | 1 |
| | 1 |
| | 1 |
| | 1 |
| | 1 |
| | 1 |
| 200 nM | |
| | 0,80794497 |
| | 0,79198009 |
| | 0,88429428 |
| | 1,01620678 |
| | 1,08387989 |
| | 1,48393262 |
| 400 nM | |
| | 0,8010854 |
| | 0,91489134 |
| | 0,86628019 |
| | 0,99070195 |
| | 0,8612235 |
| | 1,09268283 |
| | 33,4603135 |
| 800 nM | |
| | 0,87885749 |
| | 1,03222123 |
| | 0,89747081 |
| | 1,12370928 |
| | 1,08742427 |
| | 0,9502252 |
| | 26,0722405 |
