# Supplementary material for: Low androgen signaling rescues genome integrity with innate immune response by reducing fertility in humans
Source: Cell Death Dis. 2024 Jan 11;15(1):30. doi: 10.1038/s41419-023-06397-5 (PMC10784536; doi:10.1038/s41419-023-06397-5)
Supplement: Supplementary file 11 — Supplementary dataset 7 [file 41419_2023_6397_MOESM11_ESM.docx]

**Supplementary dataset 7**

**DNA alignment of TP53 gene sequencing from the gonadal tissue from individuals with DSD**

**Exon 5**

**GCT-2 vs ctrl**

GCT-2_gonad CGGAC-CGGGTGCCGGGAGGGGGTGTGGAATCAACCCACAGCCT-CACAGGGCAGGTCTT 452

||||| ||||||||||| ||||||||||||||||||||||| || |||||||||||||||

TP53_ctrl_ex5 CGGACGCGGGTGCCGGGCGGGGGTGTGGAATCAACCCACAGNCTGCACAGGGCAGGTCTT 43

GCT-2_gonad TGCCAGTTGGCAAAACATCTTGTTGAGGGCAGGATAGTAC 492

|||||||||||||||||||||||||||||||| |||||

TP53_ctrl_ex5 GGCCAGTTGGCAAAACATCTTGTTGAGGGCAGGGGAGTAC 3

**GCT-3 vs ctrl**

GCT-3_gonad 1 -----------CGCGCTGATGTTGGTCTTGTCAGCAAGATGTTTTGCCGA 39

.||.| |||.|||||||||||||| |

ctrl_TP53_ex5 1 CAGTACTCCCCTGCCC--------------TCAACAAGATGTTTTGCC-A 35

GCT-3_gonad 40 ACAGGCCAAAGA-CTGCCCTGTGCATCCGTGGGAAGAAGACAAAAACCCC 88

||.|||| |||| ||||||||||||.|.|||||..||...| |.|||||

ctrl_TP53_ex5 36 ACTGGCC-AAGACCTGCCCTGTGCAGCTGTGGGTTGATTCC--ACACCCC 82

GCT-3_gonad 89 CGCCCGGCACCCGCGGCCGGGCCAGGGCCAATCTACAAGCCGACACAAGC 138

|||||||||||||||.|||.||||.|||| ||||||||||.|.||| |||

ctrl_TP53_ex5 83 CGCCCGGCACCCGCGTCCGCGCCATGGCC-ATCTACAAGCAGTCAC-AGC 130

GCT-3_gonad 139 ACAAAAAAAAAAAAGAGGTGA-----GTTGGGGGGGGGGG---------- 173

||| ||| ||||.|.||.|..|

ctrl_TP53_ex5 131 ACA---------------TGACGGAGGTTGTGAGGCGCTGCCCCCACCAT 165

GCT-3_gonad 174 GGGCAAAAAAAAGCTGCTAAGAAAAGCGAATTTGTTTAGCAGCTGGGGCT 223

|.|| ||||||.|| |.||||| ||.|.||||||||||||

ctrl_TP53_ex5 166 GAGC--------GCTGCTCAG-ATAGCGA---TGGTGAGCAGCTGGGGC- 202

GCT-3_gonad 224 TGGA-AGAGGACAAAAAAGGGCAGGAACAAAAAAGAAAAAAAGGAAAAAC 272

|||| |||.||| |||||.||

ctrl_TP53_ex5 203 TGGAGAGACGAC-----AGGGCTGG------------------------- 222

**GCT-6 vs ctrl**

GCT-6_gonad 1 GCTACAGCAACCCCTCCACCCCAAACAAGATGTTTT-CCAATTGGCCAAA 49

||| .||.||.|..|||..||||||||||||| ||||.|||||||.

ctrl_TP53_ex5 1 ----CAG-TACTCCCCTGCCCTCAACAAGATGTTTTGCCAACTGGCCAAG 45

GCT-6_gonad 50 GACTGCCCTGT-CAACATGTGGGAATGATACACAACACCCCCGCCCGGCA 98

..||||||||| ||.| |||||| .|||| .|.||||||||||||||||

ctrl_TP53_ex5 46 ACCTGCCCTGTGCAGC-TGTGGG-TTGAT--TCCACACCCCCGCCCGGCA 91

GCT-6_gonad 99 CCCGCGATCCGCACCAAGGGGCCATCTTTAACCAGTCACAAACAAAAAAA 148

|||||| |||||.|||. ||||||||..||.||||| |||..|.|.

ctrl_TP53_ex5 92 CCCGCG-TCCGCGCCAT--GGCCATCTACAAGCAGTC----ACAGCACAT 134

GCT-6_gonad 149 AAAAAAGATTGTTTTAGGGGGGCG-TGCGCCCCACCAATAAGCCCTTGCT 197

.|...||.|||| |.|||| ||| ||||||| ||.|||.| ||||

ctrl_TP53_ex5 135 GACGGAGGTTGT------GAGGCGCTGC-CCCCACC-ATGAGCGC-TGCT 175

GCT-6_gonad 198 CAAAAATAGCGATTTTTTTAGCAACCATAGGGCCTGGAAGAAAAAAAACA 247

| |.||||||| |..|.||||.| .|||.||| ||.|.|..|||

ctrl_TP53_ex5 176 C--AGATAGCGA--TGGTGAGCAGC---TGGGGCTG---GAGAGACGACA 215

GCT-6_gonad 248 AGGGCCTAGAAACCAAGGAAAAAAAAAAAAGAACCCCCAAAAAAAGCCAA 297

.|| ||.|...||.|||.. |||||.

ctrl_TP53_ex5 216 GGG--CTGGTTGCCCAGGGT--------------CCCCAG---------- 239

**Exon 6**

**GCT-2 vs ctrl**

GCT-2_gonad CCTCTGATTCCTCACTGATTGCTCTTAGGTCTGGCCCCTCCTCAGCATCTTATCCGAGTG 81

||||||||||||||||||||||||||||||||||||||||||||||||||||||||||||

TP53_ctrl_ex6 CCTCTGATTCCTCACTGATTGCTCTTAGGTCTGGCCCCTCCTCAGCATCTTATCCGAGTG 60

GCT-2_gonad GAAGGAAATTTGCGTGTGGAGTATTTGGATGACAGAAACACTTTTCGACATAGTGTGGTG 141

||||||||||||||||||||||||||||||||||||||||||||||||||||||||||||

TP53_ctrl_ex6 GAAGGAAATTTGCGTGTGGAGTATTTGGATGACAGAAACACTTTTCGACATAGTGTGGTG 120

GCT-2_gonad -TGCCCTATG-GCCGCCTGAG 160

||||||||| ||||||||||

TP53_ctrl_ex6 GTGCCCTATGAGCCGCCTGAG 141

**GCT-3 vs ctrl**

GCT-3_gonad CCTCTGATTCCTCACTGATTGCTCTTAGGTCTGG-CCCTCCTCAGCATCTTATCCGAGTG 200

|||||||||||||||||||||||||||||||||| |||||||||||||||||||||||||

TP53_ctrl_ex6 CCTCTGATTCCTCACTGATTGCTCTTAGGTCTGGCCCCTCCTCAGCATCTTATCCGAGTG 60

GCT-3_gonad GAAGGAAATTTGCGTGTGGAGTATTTGGATGACAGAAACACTTTTCGACATAGGTGTGGT 260

|||||||||||||||||||||||||||||||||||||||||||||||||||| |||||||

TP53_ctrl_ex6 GAAGGAAATTTGCGTGTGGAGTATTTGGATGACAGAAACACTTTTCGACATA-GTGTGGT 119

GCT-3_gonad GGTGCCCTATG-G-CGCCTGAGGTCTTGTTT 289

||||||||||| | |||||||||||| ||||

TP53_ctrl_ex6 GGTGCCCTATGAGCCGCCTGAGGTCTGGTTT 150

**GCT-6 vs ctrl**

GCT-6_gonad TTAACCCCTCCTCCCAGAGACCCCAGTTGCAAACCAGACCTCAGGCGGCTCATAGGGCAC 150

||||||||||||||||||||||||||||||||||||||||||||||||||||||||||||

TP53_ctrl_ex6 TTAACCCCTCCTCCCAGAGACCCCAGTTGCAAACCAGACCTCAGGCGGCTCATAGGGCAC 121

GCT-6_gonad CACCACACTATGTCGAAAAGTGTTTCTGTCATCCAAATACTCCACACGCAAATTTCCTTC 210

||||||||||||||||||||||||||||||||||||||||||||||||||||||||||||

TP53_ctrl_ex6 CACCACACTATGTCGAAAAGTGTTTCTGTCATCCAAATACTCCACACGCAAATTTCCTTC 61

GCT-6_gonad GACTCGGATAAGG-GCTGAGGAGGG 234

||||||||||| |||||||||||

TP53_ctrl_ex6 CACTCGGATAAGATGCTGAGGAGGG 36

**Exon 7**

**GCT-2 vs ctrl**

GCT-2_gonad GCCGTCATCTTGGGCCTGTGTAATCTCCTAGGTTGGTTCTGAGTCTACCACCATCCACTA 66

||| ||||||||||||||||| |||||||||||||| ||||| | |||||||||||||||

TP53_ctrl_ex7 GCC-TCATCTTGGGCCTGTGTTATCTCCTAGGTTGGCTCTGACTGTACCACCATCCACTA 78

GCT-2_gonad CAACTACATGTGTAACAGTTCCTGCATGGGCGGCATGAACCGGAGGCCCATCCTCACCAT 126

||||||||||||||||||||||||||||||||||||||||||||| ||||||||||||||

TP53_ctrl_ex7 CAACTACATGTGTAACAGTTCCTGCATGGGCGGCATGAACCGGAGGCCCATCCTCACCAT 138

GCT-2_gonad CATCACACTGGAAGACTCCAGGTCAGGAGCCACTTGCCACCCTGCACACTGGCCTGCTGT 186

||||||||||||||||||||||||||||||||||||||||||||||||||||||||||||

TP53_ctrl_ex7 CATCACACTGGAAGACTCCAGGTCAGGAGCCACTTGCCACCCTGCACACTGGCCTGCTGT 198

GCT-2_gonad -CTCCAGCCTCTGCTTGCCTCTGACCCCT 214

| ||||||||||||||||||||||||||

TP53_ctrl_ex7 GCCCCAGCCTCTGCTTGCCTCTGACCCCT 22

**GCT-3 vs ctrl**

GCT-3_gonad GGCGTCATCTTGGGCCTGTGTTATCTCCTAGGTTGGCTCTGACTGTACCACCATCCACTA 67

||| |||||||||||||||||||||||||||||||||||||| |||||||||||||||||

TP53_ctrl_ex7 GGCCTCATCTTGGGCCTGTGTTATCTCCTAGGTTGGCTCTGACTGTACCACCATCCACTA 78

GCT-3_gonad CAACTACATGTGTAACAGTTCCTGCATGGGCGGCATGAACCGGAGGCCCATCCTCACCAT 126

|||||||||||||||||||||||||||||||||||||||||||| |||||||||||||||

TP53_ctrl_ex7 CAACTACATGTGTAACAGTTCCTGCATGGGCGGCATGAACCGGAGGCCCATCCTCACCAT 138

GCT-3_gonad CATCACACTGGAAGACTCCAGGTCAGGAGCCACTTGCCACCCTGCACACTGGCCTGCTGT 186

||||||||||||||||||||||||||||||||||||||||||||||||||||||||||||

TP53_ctrl_ex7 CATCACACTGGAAGACTCCAGGTCAGGAGCCACTTGCCACCCTGCACACTGGCCTGCTGT 198

GCT-3_gonad -CCCCAGCCTCTGCTTGCCTCTTACCCCT 214

||||||||||||||||||||| ||||||

TP53_ctrl_ex7 GCCCCAGCCTCTGCTTGCCTCTGACCCCT 227

**GCT-6 vs ctrl**

GCT-6_gonad 1 ---------------AGCTTGACC-----TCT-GCCCATCCGCAGCACCT 29

.|.|||..| ||| ||||.|||.|||||.||

TP53_ctrl_ex7 1 CCTCTGATTCCTCACTGATTGCTCTTAGGTCTGGCCCCTCCTCAGCATCT 50

GCT-6_gonad 30 TACCCGAGGGGAAAGGAAATTTGCGTGTGGAGTATTTGGCATGACAGGAA 79

||.|||||.|| ||||||||||||||||||||||||||| ||||||| |

TP53_ctrl_ex7 51 TATCCGAGTGG-AAGGAAATTTGCGTGTGGAGTATTTGG-ATGACAG--A 96

GCT-6_gonad 80 AACACAATTTCGACATAGTGTGGTGGTGCCCTTATGAGCCGCCTGTTTTT 129

||||| .|||||||||||||||||||||||| |||||||||||||...|

TP53_ctrl_ex7 97 AACAC-TTTTCGACATAGTGTGGTGGTGCCC-TATGAGCCGCCTGAGGT- 143

GCT-6_gonad 130 CCTGGCCTCGCAAATGAAAAAAAATAGGAGGAGGGGGGGGGGGGGG---- 175

|||| .|.||||.|| .......|.||||||||||....|||.||

TP53_ctrl_ex7 144 -CTGG-TTTGCAACTG-GGGTCTCTGGGAGGAGGGGTTAAGGGTGGTTGT 190

GCT-6_gonad 176 --GAGAAACAACCGGGGAGCA--CGAGGGGTTT-----------TTGTTT 210

|.|...|..|.||.||||| .|.||||.|| ||.|||

TP53_ctrl_ex7 191 CAGTGGCCCTCCAGGTGAGCAGTAGGGGGGCTTTCTCCTGCTGCTTATTT 240

**Exon 8-9**

**GCT-2 vs ctrl**

GCT-2_gonad TCTCTTTTCCTATCCTGAGTAGTGGTAATCTACTGGGACGGAACAGCTTTGAGGTGCGTG 131

||||||||||||||||||||||||||||||||||||||||||||||||||||||||||||

TP53_ctrl_exon8-9 TCTCTTTTCCTATCCTGAGTAGTGGTAATCTACTGGGACGGAACAGCTTTGAGGTGCGTG 60

GCT-2_gonad TTTGTGCCTGTCCTGGGAGAGACCGGCGCACAGAGGAAGAGAATCTCCGCAAGAAAGGGG 191

||||||||||||||||||||||||||||||||||||||||||||||||||||||||||||

TP53_ctrl_exon8-9 TTTGTGCCTGTCCTGGGAGAGACCGGCGCACAGAGGAAGAGAATCTCCGCAAGAAAGGGG 120

GCT-2_gonad AGCCTCACCACGAGCTGCCCCCAGGGAGCACTAAGCGAGGTAAGCAAGCAGGACAAGAAG 251

||||||||||||||||||||||||||||||||||||||||||||||||||||||||||||

TP53_ctrl_exon8-9 AGCCTCACCACGAGCTGCCCCCAGGGAGCACTAAGCGAGGTAAGCAAGCAGGACAAGAAG 180

GCT-2_gonad CGGTGGAGGAGACCAAGGGTGCAGTTATGCCTCAGATTCACTTTTATCACCTTTCCTTGC 311

||||||||||||||||||||||||||||||||||||||||||||||||||||||||||||

TP53_ctrl_exon8-9 CGGTGGAGGAGACCAAGGGTGCAGTTATGCCTCAGATTCACTTTTATCACCTTTCCTTGC 240

GCT-2_gonad CTCTTTCCTAGCACTGCCCAACAACACCAGCTCCTCTCCCCAGCCAAAGAAGAAACCACT 371

||||||||||||||||||||||||||||||||||||||||||||||||||||||||||||

TP53_ctrl_exon8-9 CTCTTTCCTAGCACTGCCCAACAACACCAGCTCCTCTCCCCAGCCAAAGAAGAAACCACT 300

GCT-2_gonad GGATGGAGAATATTTCACCCTTCAGGTACTAAGTCTTGGGACCTCTTAT 420

|||||||||||||||||||||||||||||||||||||||||||||||||

TP53_ctrl_exon8-9 GGATGGAGAATATTTCACCCTTCAGGTACTAAGTCTTGGGACCTCTTAT 349

**GCT-3 vs ctrl**

GCT-3_gonad TCTCTTTTCCTATCCTGAGTAGTGGTAATCTACTGGGACGGAACAGCTTTGAGGTGCGTG 132

||||||||||||||||||||||||||||||||||||||||||||||||||||||||||||

TP53_ctrl_exon8-9 TCTCTTTTCCTATCCTGAGTAGTGGTAATCTACTGGGACGGAACAGCTTTGAGGTGCGTG 60

GCT-3_gonad TTTGTGCCTGTCCTGGGAGAGACCGGCGCACAGAGGAAGAGAATCTCCGCAAGAAAGGGG 192

||||||||||||||||||||||||||||||||||||||||||||||||||||||||||||

TP53_ctrl_exon8-9 TTTGTGCCTGTCCTGGGAGAGACCGGCGCACAGAGGAAGAGAATCTCCGCAAGAAAGGGG 120

GCT-3_gonad AGCCTCACCACGAGCTGCCCCCAGGGAGCACTAAGCGAGGTAAGCAAGCAGGACAAGAAG 252

||||||||||||||||||||||||||||||||||||||||||||||||||||||||||||

TP53_ctrl_exon8-9 AGCCTCACCACGAGCTGCCCCCAGGGAGCACTAAGCGAGGTAAGCAAGCAGGACAAGAAG 180

GCT-3_gonad CGGTGGAGGAGACCAAGGGTGCAGTTATGCCTCAGATTCACTTTTATCACCTTTCCTTGC 312

||||||||||||||||||||||||||||||||||||||||||||||||||||||||||||

TP53_ctrl_exon8-9 CGGTGGAGGAGACCAAGGGTGCAGTTATGCCTCAGATTCACTTTTATCACCTTTCCTTGC 240

GCT-3_gonad CTCTTTCCTAGCACTGCCCAACAACACCAGCTCCTCTCCCCAGCCAAAGAAGAAACCACT 372

||||||||||||||||||||||||||||||||||||||||||||||||||||||||||||

TP53_ctrl_exon8-9 CTCTTTCCTAGCACTGCCCAACAACACCAGCTCCTCTCCCCAGCCAAAGAAGAAACCACT 300

GCT-3_gonad GGATGGAGAATATTTCACCCTTCAGGTACTAAGTCTTGGGACCTCTTATAAAGT 426

||||||||||||||||||||||||||||||||||||||||||||||||| ||||

TP53_ctrl_exon8-9 GGATGGAGAATATTTCACCCTTCAGGTACTAAGTCTTGGGACCTCTTATCAAGT 354

**GCT-6 vs ctrl**

GCT-6_gonad TCTCTTTTCCTATCCTGAGTAGTGGTAATCTACTGGGACGGAACAGCTTTGAGGTGCGTG 128

|||||||||||||||||||||||||||||| |||||||||||||||||||||||||||||

TP53_ctrl_exon8-9 TCTCTTTTCCTATCCTGAGTAGTGGTAATCTACTGGGACGGAACAGCTTTGAGGTGCGTG 60

GCT-6_gonad TTTGTGCCTGTCCTGGGAGAGACCGGCGCACAGAGGAAGAGAATCTCCGCAAGAAAGGGG 188

||||||||||||||||||||||||||||||||||||||||||||||||||||||||||||

TP53_ctrl_exon8-9 TTTGTGCCTGTCCTGGGAGAGACCGGCGCACAGAGGAAGAGAATCTCCGCAAGAAAGGGG 120

GCT-6_gonad AGCCTCACCACGAGCTGCCCCCAGGGAGCACTAAGCGAGGTAAGCAAGCAGGACAAGAAG 246

||||||||||||||||| |||||||||||||||||||||||||| |||||||||||||||

TP53_ctrl_exon8-9 AGCCTCACCACGAGCTGCCCCCAGGGAGCACTAAGCGAGGTAAGCAAGCAGGACAAGAAG 180

GCT-6_gonad CGGTGGAGGAGACCAAGGGTGCAGTTATGCCTCAGATTCACTTTTATCACCTTTCCTTGC 306

||||||||||||||||||||||||||||||||||||||||||||||||||||||||||||

TP53_ctrl_exon8-9 CGGTGGAGGAGACCAAGGGTGCAGTTATGCCTCAGATTCACTTTTATCACCTTTCCTTGC 240

GCT-6_gonad CTCTTTCCTAGCACTGCCCAACAACACCAGCTCCTCTCCCCAGCCAAAGAAGAAACCACT 365

|||||||||| |||||||||||||||||||||||||||||||||||||||||||||||||

TP53_ctrl_exon8-9 CTCTTTCCTAGCACTGCCCAACAACACCAGCTCCTCTCCCCAGCCAAAGAAGAAACCACT 300

GCT-6_gonad GGATGGAGAATATTTCACCCTTCAGGTACTAAGTCTTGGGACCTCTTA 413

||||||||||||||||||||||||||||||||||||||||||||||||

TP53_ctrl_exon8-9 GGATGGAGAATATTTCACCCTTCAGGTACTAAGTCTTGGGACCTCTTA 348
